# A Resilient Distributed Pareto-Based PSO for Edge-UAVs Deployment Optimization in Internet of Flying Things

**DOI:** 10.3390/s25216554

**Published:** 2025-10-24

**Authors:** Sabrina Zerrougui, Sofiane Zaidi, Carlos T. Calafate

**Affiliations:** 1 Department of Mathematics and Computer Science, Research Laboratory on Computer Science’s Complex Systems (ReLa(CS)^2^), University of Oum El Bouaghi, Oum El Bouaghi 04000, Algeria; zaidi.sofiane@univ-oeb.dz; 2 Department of Computer Engineering (DISCA), Universitat Politècnica de València, 46022 Valencia, Spain

**Keywords:** particle swarm optimization, UAV deployment, multi-objective optimization, epsilon constraint, Pareto archive, weighted sum, Internet of Flying Things

## Abstract

Particle Swarm Optimization (PSO) has been widely employed to optimize the deployment of Unmanned Aerial Vehicles (UAVs) in various scenarios, particularly because of its efficiency in handling both single and multi-objective optimization problems. In this paper, a framework for optimizing the deployment of edge-enabled UAVs using Pareto-PSO is proposed for data collection scenarios in which UAVs operate autonomously and execute onboard distributed multi-objective PSO to maximize the total non-overlapping coverage area while minimizing latency and energy consumption. Performance evaluation is conducted using key indicators, including convergence time, throughput, and total non-overlapping coverage area across bandwidth and swarm-size sweeps. Simulation results demonstrate that the Pareto-PSO consistently attains the highest throughput and the largest coverage envelope, while exhibiting moderate and scalable convergence times. These results highlight the advantage of treating the objectives as a vector-valued objective in Pareto-PSO for real-time, scalable, and energy-aware edge-UAV deployment in dynamic Internet of Flying Things environments.

## 1. Introduction

The Internet of Flying Things (IoFT) represents a multi-layered network architecture that integrates Unmanned Aerial Vehicles (UAVs), commonly known as drones, as the primary physical entities in Flying Ad Hoc Network (FANET) environments, in conjunction with Internet of Things (IoT) technology [1]. This integration facilitates real-time data exchange, autonomous coordination, and effective management of low-altitude airspace [2].

Given their versatile functionalities and rapid deployment, UAVs are increasingly used to collect real-time data from hard-to-reach locations. A prominent application is providing wireless coverage to distributed ground users (GUs), where UAVs can be deployed as flying base stations (FBSs), cellular base stations, or Wi-Fi access points to extend connectivity in areas lacking traditional communication infrastructure [3,4,5].

UAV deployment refers to positioning and operating UAVs for specific tasks across a wide range of civil and military application domains [6]. After disasters, UAVs can serve as mobile communication systems to connect GUs and survivors [7], acting as first-response platforms that support rescue efforts [8]. Furthermore, UAVs can act as a platform for the delivery of commercial services to consumers [9]. In surveillance and monitoring missions, effective UAV deployment plays a crucial role in facilitating data collection and improving the quality of coverage for the targeted areas of interest [10]. In addition, within network reconstruction scenarios, UAVs can function as aerial relays to re-establish wireless links and reconnect isolated ground networks, particularly in disaster situations where fixed ad hoc networks have been severely compromised [11].

By positioning edge servers closer to the users, Mobile Edge Computing (MEC) reduces network congestion that often occurs with traditional cloud-based systems. This local processing leads to accelerated data processing that minimizes delays, leading to better performance and better Quality of Service (QoS), especially in tasks requiring real-time responses [2], collecting data missions, emergency communication, and rescue operations.

In the Internet of Drones (IoD), edge computing technology enhances the capabilities of both fog and cloud computing to support real-time IoT applications. By processing a portion of UAV data locally on edge IoD devices, the edge layer reduces the computing load without relying on fog or cloud interventions. This shift toward edge-based data processing and storage promotes significant latency improvements [12]. UAVs can be considered as edge nodes capable of offering computing services to GUs, with the aim of minimizing the average task response time by jointly optimizing UAV deployment and computation offloading [3].

Optimizing UAV deployment is a key area of ongoing research [13]. Deployment studies typically prioritize latency, coverage, and energy consumption, especially under rapid deployment and real-time decision making. These objectives directly shape network efficiency, responsiveness, and sustainability. Latency governs end-to-end data transfer and processing speed; coverage defines the served area; and energy consumption critically determines UAV operational lifetime [13]. Therefore, it is crucial to reduce peak energy consumption before service commencement [5]. Additional metrics (e.g., QoS variants, link reliability, security) are important but often increase model complexity. In mission-critical contexts, prioritizing latency, coverage, and energy preserves tractability for timely decisions.

For instance, the Particle Swarm Optimization (PSO) algorithm is a lightweight metaheuristic noted for rapid convergence, efficient search, and adaptability; it effectively handles landscapes with multiple local optima [14].

To adopt a consistent and effective metric for multi-objective edge-UAV deployment based on the well-known PSO metaheuristic, this paper proposes and implements a Pareto-PSO framework for edge-UAV deployment and compares it against two PSO-based scalarization baselines. These include the following:

- Pareto-PSO with external archive:treats the objectives as a vector and maintains an external Pareto archive of non-dominated solutions to approximate the Pareto front.
- Weighted-sum PSO: scalarizes the multi-objective problem using predefined weights to optimize a single composite objective.
- Epsilon-constrained PSO: rotates the primary objective by optimizing one objective while imposing the remaining objectives as constraints.

The combination of scalarization and vector-based approaches has been rarely examined in prior work, and our comparison of Pareto-PSO with PSO-based scalarization baselines shows that fitness function design—scalar versus vector—materially affects the performance of distributed PSO deployment strategies in data collection and emergency response missions. These results guide the selection of methods that achieve real-time, scalable, and efficient deployment trade-offs for edge-UAVs operating in dynamic environments. Because distributed algorithms operate solely on locally available data, they enable informed decision making with minimal information exchange, thereby reducing communication overhead and latency [4]. This rationale motivates distributing the PSO metaheuristic across the edge-UAV swarm.

Furthermore, in this paper, we aim to identify the strengths, limitations, and performance characteristics of using scalarization techniques to convert a multi-objective edge-UAV deployment optimization problem into a single-objective one. In addition, our goal is also to use a vectorization technique that combines multiple objectives directly. The goal is to assess how scalarization compares to directly handling the problem as a vector of multiple objective functions, particularly in terms of convergence speed, throughput, and coverage efficiency. The evaluation is based on a scenario in which a swarm of edge-UAVs are deployed over an area of interest to gather and receive various types of information, such as recorded videos, voice messages, or text messages, from distributed GUs and then offload the collected data to a fog computing node. In this work, resilience refers to the framework’s algorithmic robustness and adaptability to user mobility and dynamic edge network conditions, rather than explicit tolerance to UAV or communication failures.

The remainder of this paper is organized as follows. Section 2 reviews the related work. Section 3 presents the basic concepts and techniques used. Section 4 describes the proposed system model and details the problem formulation. Section 5 presents and discusses the simulation results and performance evaluation. Section 6 concludes the paper and discusses future research directions.

## 2. Related Work

Many meta-heuristics and techniques have been implemented for single-objective optimization and even for multi-objective optimization in UAV deployment, such as Genetic Algorithm (GA), PSO, Ant Colony Optimization (ACO), and Evolutionary algorithms, among others.

In this section, we cover two subsections. The first subsection presents an overview of the proposed and designed UAV deployment algorithms to address the specific application challenges that arise when positioning and operating UAVs for different tasks, including overcoming technical UAV constraints such as energy consumption and resource limitations, along with practical complexities of real-world deployments such as cost effectiveness in dynamic and often unpredictable environments. The second subsection then discusses studies that investigate the use of the PSO algorithm to optimize the UAV deployment problem.

### 2.1. Overview of UAV Deployment Algorithms

Fan et al. [6] aimed to reduce the probability of UAV detection by adversary systems during real-time monitoring missions in tactical battlefield scenarios, allowing UAVs to identify potential targets. They proposed a Distributed Virtual Force Motion Control Algorithm (DVFMC) to achieve this goal. The algorithm operates without requiring global information and relies on local coverage of the path-demand area. MATLAB simulations validated the efficiency of the proposed method in reducing the probability of detection. The resulting UAV network was proved to be effective for real-time operation with high reliability, making it well suited for battlefield monitoring applications.

Rhodes et al. [7] present an advanced UAV deployment approach for autonomous gas source search and localization in unknown GPS-denied environments. Their system leverages a UAV platform that integrates the Dual Control for Exploitation and Exploration (DCEE) utility function with a Rapidly exploring Random Tree (RRT)-based path planning algorithm. This combination is central to the UAV deployment strategy, enabling the UAV to autonomously map its environment, avoid obstacles, and make real-time decisions about where to sample next. The proposed approach ensures that UAVs can explore unknown environments safely, efficiently, and intelligently, dynamically adapting their deployment to maximize the effectiveness of the search and estimation process, thereby addressing real-world challenges in hazardous source locations.

Huang and Savkin [10] proposed a reactive, decentralized, and collision-free 3D deployment algorithm for surveillance and monitoring applications. The algorithm aims to maximize the quality of coverage (QoC) of targets by deploying UAVs in a continuous 3D space and navigating each UAV based on local measurements rather than global information. MATLAB simulations demonstrate the effectiveness of the algorithm in achieving the desired coverage performance.

Liu et al. [15] proposed two centralized UAV deployment algorithms. The first algorithm, UAV Deployment Algorithm Degree and Distance (UDA-DD), uses the distance between the base stations and the user devices, along with the user degree, as metrics to build and update service connections. In contrast, the second algorithm, UAV Deployment Algorithm Degree (UDA-DS), focuses on maintaining the QoS between base stations and user devices based on the user degree and SINR between users and base stations. Both algorithms seek to formulate the full communication coverage problem with objectives such as minimizing the number of UAVs deployed, ensuring the quality of service, and guaranteeing network robustness. However, both algorithms differ in how they provide coverage in two scenarios. The first scenario addresses the absence of terrestrial static base stations, while the second considers situations where static base stations exist but fail to deliver sufficient communication coverage to all users. Simulations were performed using MATLAB R2015b, and the results demonstrated that both UDA-DD and UDA-DS improved the QoS and ensured robust network performance during UAV deployment. However, since these approaches are centralized, they may not be suitable for large-scale or dynamic environments.

In [16], Trotta et al. propose a distributed framework for an aerial mesh deployment system named the aErial Local Positioning System for Emergency (ELAPSE), where UAVs are used as mobile base stations. The authors deployed these UAVs to provide emergency services, maintain coverage, establish connectivity between isolated rescue teams, and assist affected individuals in areas lacking infrastructure. The UAV swarm operates without relying on Global Positioning System (GPS) signals; instead, it uses a combination of local and cooperative positioning techniques. The results of OMNeT++ simulations demonstrate the effectiveness of the proposed framework.

Lee and Friderikos [17] investigated the use of UAVs as FBSs that can be associated with different macro-base stations to address trajectory optimization in multi-cell wireless networks. The proposed UAV deployment algorithm, based on Mixed Integer Linear Programming (MILP), introduces a novel optimization framework that determines the optimal trajectory of FBSs to minimize the Total Travel Time (TTT) while serving GUs across multiple cells.

In [18], Miao et al. explored the use of UAVs as MEC nodes to improve the computing and storage capacity of the IoT industry and smart city applications during natural disasters. They propose a UAV deployment method called Ground-Air Controlled Global and Local Path Planning for UAV Swarm-MEC Offloading Strategy (GAGLPP), which aims to achieve three objectives: maximize offloading services, minimize path length, and maximize energy efficiency. The experimental results validate the effectiveness of the GAGLPP strategy.

In [19], Huang and Savkin investigated the deployment of UAV-mounted base stations after disasters or during temporary events to provide optimal coverage for a set of GUs. Their algorithm aims to minimize the distance between UAVs and GUs while also maintaining connectivity with fixed base stations. UAVs are deployed at different altitudes to serve GUs, ensuring strong signal quality in both Line-Of-Sight (LOS) and Non-Line-Of-Sight (NLOS) conditions. The proposed decentralized deployment algorithm assumes direct air-to-ground LOS communication, allowing each UAV to determine its movement based solely on local information.

Zhang et al. [20] introduce a novel deep reinforcement learning (DRL) framework to optimize the deployment of tethered UAVs in millimeter-Wave (mmWave) Integrated Access and Backhaul (IAB) networks, with a particular focus on dense urban environments. The proposed approach, based on the TD3-MB algorithm, jointly addresses three main objectives: minimizing the number of required UAVs and terrestrial base stations, optimizing the hovering positions of UAVs, and configuring the multi-hop backhauling topology. The deployment problem is formulated as a constrained optimization task, where the goal is to reduce the overall deployment cost while ensuring that all client nodes are connected to the network core and meet strict quality-of-service requirements. By modeling the environment as a Markov Decision Process, and by applying a non-cumulative reward criterion via the max-Bellman optimality equation, the framework effectively learns optimal deployment and routing strategies. The study demonstrates that this method adapts efficiently to complex urban scenarios with varying blockage densities, as validated through case studies in cities in Asia, Europe, and North America. The results highlight the framework’s ability to overcome key challenges such as line-of-sight constraints, deployment cost, power limitations, and scalability, ultimately enabling robust and cost-effective UAV-based network deployments in practical settings.

Qi et al. [21] propose an optimization strategy that addresses the problem of minimizing the latency among multi-rotor surveillance UAVs involved in video transmission to a relay UAV for maritime search and rescue operations. The problem is decomposed into three sub-problems: (i) offloading decision optimization, (ii) relay UAV position optimization, and (iii) association optimization between surveillance UAVs and targets. The proposed approach effectively reduces the maximum latency and balances the delay across all surveillance UAVs, enabling faster and more fair rescue decision making. However, the study assumes known target locations and static environmental conditions, and its computational complexity may limit scalability for large-scale or highly dynamic scenarios.

Table 1 provides a comprehensive overview of some of the latest and significant studies on UAV deployment algorithms, evaluating them according to three essential criteria: the type of UAV system used, the primary application objective, and the architectural approach to decision making and information usage. The criteria are described as follows:

- The first criterion considers the type of UAV; for instance, some works focus on deploying single UAVs as flying base stations (FBSs) or relay nodes, emphasizing areas such as coverage maximization and seamless backhaul integration. In contrast, other studies leverage UAV swarms to achieve robust mesh networking or adopt UAV-based mobile edge computing (MEC) architectures to support distributed, real-time data processing in dynamic industrial environments.
- The second criterion focuses on the application objective of each deployment algorithm, as presented in the respective studies. Beyond technical specifications, the table highlights how each algorithm’s objective is tailored to specific operational contexts. Coverage-focused algorithms are particularly relevant for disaster response and rural connectivity, where rapid deployment and reliable service are priorities. Meanwhile, connectivity-driven approaches address the complexities of maintaining robust inter-UAV links in challenging or contested environments. The inclusion of MEC-oriented algorithms underscores the growing importance of computational offloading at the network edge.
- The third criterion is an assessment of the algorithmic architecture: whether control is centralized, or distributed, and whether deployment decisions are based on global or local information.

**Table 1:** Comparison of UAV deployment algorithms.

Paper	Method	UAV as			Objective				Architecture			
		FBS	Swarm	MEC	Connectivity	Coverage	Computation	Optimal Path	Centralized	Distributed	Local Info	Global Info
Fan et al. [6]	DVFMC		✓		✓	✓				✓	✓	
Huang and Savkin [10]	GPS-Free Decentralized Flocking		✓		✓	✓	✓	✓				
Lui et al. [15]	UDA-DD and UDA-DS	✓				✓			✓			
Trotta et al. [16]	ELAPSE	✓			✓	✓				✓		
Lee and Friderikos [17]	GAGLPP	✓										✓
Miao et al. [18]	Deep RL-based swarm path planning			✓			✓					
Huang and Savkin [19]	Reactive Collision Free 3D	✓			✓	✓				✓	✓	

### 2.2. Literature Review on UAV Deployment Based on PSO Meta-Heuristic

Chen et al. [3] proposed a hybrid PSO-based method called the PSO-Genetic Algorithm-Greedy (PSO-GA-G) to jointly optimize UAV deployment and computation offloading in multi-UAV-enabled MEC systems. The method adopts a two-layer solution: The outer layer combines PSO and GA to determine optimal UAV positions, while the inner layer uses a greedy algorithm to decide which tasks received from mobile devices should be offloaded. The approach aims to minimize the average task response time in a dynamic environment but faces constraints on computational complexity, especially with GA, real-world modeling, and energy awareness.

In [4], Hydher et al. focused on optimizing UAV deployment in disaster-affected areas to maximize spectral efficiency while minimizing energy consumption. The problem was divided into two sub-problems: (i) horizontal placement and user assignment and (ii) altitude optimization. For the former, they applied K-means clustering combined with the stable marriage algorithm. For altitude optimization, they proposed two methods: a basic exhaustive search and a more efficient PSO meta-heuristic. The proposed approach enabled significant energy savings and achieves higher spectral efficiency, especially in dense networks. However, the study assumed static user locations and centralized control, which can limit its adaptability and practicality in highly dynamic or large-scale disaster scenarios.

Lin et al. [13] proposed an enhanced optimization algorithm called Double Self-Limiting PSO (DSLPSO), which is designed to improve UAV energy efficiency in data collection missions for IoT scenarios. The algorithm focuses on determining the optimal number and locations of UAV stopping points, minimizing the total energy consumption of both the UAV and IoT devices. By planning more energy-efficient trajectories, DSLPSO extends mission duration and outperforms existing algorithms in terms of energy savings and adaptability to varying mission sizes. Notably, the method introduces Self-Limiting Radius and Multiple Simulated Annealing (SLRMSA) mechanisms to balance global and local search capabilities. However, the study is limited by its focus on single-UAV scenarios, fixed flight altitude, and idealized simulation environments, which may affect scalability and practical deployment in more complex settings.

Liu et al. [14] proposed a hybrid approach named Tent-PSO that combines the standard PSO technique with tent chaotic mapping to optimize drone paths. The approach aims to maximize throughput, increase user coverage, maintain link quality, and enhance global search efficiency in real-time, with drones deployed as communication relays to provide reliable connectivity for mobile GUs. By replacing random initialization of the positions of particles with tent mapping, the algorithm prevents particles from inefficient exploration and reduces the risk of getting trapped in local optima. The Tent-PSO approach is difficult to apply practically, since its outcomes rely heavily on the parameters that are chosen, and it does not evaluate real-life scenarios.

Abdel-Razeq et al. [22] investigated the joint optimization of 3D UAV deployment and a transmit power allocation strategy for a UAV-enabled uplink Non-Orthogonal Multiple Access (UL-NOMA) network, aiming to maximize the ergodic sum rate. For the UAV deployment stage, they applied the PSO meta-heuristic to determine the optimal 3D positioning of UAVs. The objective is to minimize the aggregate path loss between UAVs and GUs, thus ensuring efficient data collection. The study assumes perfect channel conditions and disregards guards user movement, UAV energy limits, and signal processing errors. Because it streamlines user pairing and leaves out important issues in large, real-world networks, its conclusions might not be immediately applicable to actual implementation.

In [23], Xuefeng Chen et al. proposed a hybrid intelligent coverage algorithm called the PSO–Virtual Repulsive Force Firefly Algorithm (PSO-VRFFA) to optimize UAV-mounted base stations. In this approach, the PSO algorithm serves as the general framework (for a global search, by exploring the search space for promising UAV positions), while the VFA component phase fine-tunes UAV locations to maximize user coverage and minimize overlap between UAVs. Although PSO-VFA achieves improved user coverage and faster convergence, it is constrained in terms of scalability and practical applicability in a large, dynamic, or resource-constrained environment.

Rasouli [24] aimed to use the PSO algorithm to determine the optimal placement of drones deployed as aerial base stations in crowded user environments. The objective is to maximize user coverage using the minimum number of drones while considering energy consumption as a constraint. The study demonstrates that the PSO-based approach enables the rapid and flexible deployment of UAVs, improves energy efficiency and ensures stable and reliable connections for users. However, it is limited by its reliance on an idealized simulation environment with randomly distributed users and fixed altitudes, which may not fully represent real-world conditions or address additional practical concerns such as backhaul connectivity and experimental validation.

Ibraiwish et al. [25] proposed a framework to optimize the trajectories of visible light communication-enabled UAVs by formulating a Multi-objective Optimization Problem (MOP) that jointly maximizes the user sum rate and rate fairness while minimizing UAV power consumption. The optimization is performed using the PSO algorithm with enhanced particle initialization. The advantages of the proposed method are its ability to dynamically adapt UAV trajectories to user mobility, achieve significant improvements in sum rate and user coverage, and explicitly account for the dominant energy cost of UAV propulsion. However, the study is limited by its reliance on predetermined user mobility patterns, simplified (obstacle-free) simulation environments, and potential scalability challenges as the number of UAVs and users increases.

Li et al. [26] proposed a Multi-Objective Particle Swarm Optimization (MOPSO) framework for the deployment of UAVs in multi-hop Flying Ad Hoc Networks (FANETs), supporting emergency vehicular communications. Their method formulates UAV placement as a multi-objective optimization problem that simultaneously minimizes deployment cost, networking time, and hop count, facilitating rapid and reliable relay construction between stranded vehicles and infrastructure. Utilizing terrain-aware simulations incorporating obstacles and realistic environmental factors, the MOPSO algorithm effectively identifies relay node positions that maintain stable inter-UAV links while operating within the communication range. The optimizer employs Pareto dominance principles to generate a diverse set of trade-off solutions representing different balances among conflicting objectives. A key innovation is the adoption of a branching-node selection strategy, which strategically reduces overall deployment time and cost, particularly in asymmetric network topologies. Although the approach exhibits strong adaptability to dynamic real-world conditions, the authors note that performance may be sensitive in scenarios with uniform target distributions, and underlying assumptions such as simplified channel and mobility models may restrict direct applicability to highly complex environments.

Table 2 presents a comparison between the related works listed in Section 2.2; it details UAV deployment based on the PSO meta-heuristic and compares them to our proposed PSO usage approach. This comparison is based on the following three criteria:

- The first criterion considers the most common optimization objectives; it includes maximizing user coverage, as well as minimizing energy consumption while maximizing energy efficiency during missions or minimizing latency.
- The second criterion focuses on how the PSO algorithm is used, whether it is implemented as a standard approach or in variants for the UAV deployment scenario.
- The third criterion considers the UAV type involved; most studies deploy rotary-wing UAVs as aerial base stations or relays, and some are deployed with specialized roles such as flying access points or UAV-enabled mobile edge computing systems (serving as edge servers).
- Most of the works in this table prefer to solve objectives either in (i) sequential, separate stages by using different techniques for different sub-problems or (ii) aggregate multiple objectives into a single function for standard PSO optimization. A truly simultaneous multi-objective optimization with PSO is only seen in the work of Ibraiwish et al. [25] and in the work of Li et al. [26]. Our work proposes a true multi-objective PSO framework, including Pareto-PSO in a distributed manner, allowing simultaneous optimization of coverage, latency, and energy for edge-UAV network.

**Table 2:** Comparison of UAV deployment works based on PSO meta-heuristic.

Paper	Objectives				Method		UAV Types				Objectives Handling Strategy
	Energy	Coverage	Latency	Throughput	PSO	PSO-Combined	MEC	AP	BS	Relay	
[3]			✓			✓	✓				Sequential: deployment PSO+GA, offloading greedy
[4]	✓				✓				✓		Two-stage pipeline: placement via K-means/stable marriage, altitude via standard PSO
[13]	✓					✓					Single-objective (energy-focused)
[14]		✓		✓		✓				✓	Single-objective (combined metric of multiple goals)
[22]				✓		✓			✓		Single-objective (maximize ergodic sum rate)
[23]		✓				✓			✓		Single-objective (maximize coverage, minimize overlap via weighted sum)
[24]	✓	✓			✓				✓		Single-objective (coverage primary, energy as constraint)
[25]	✓				✓			✓			Multi-objective Pareto dominance (simultaneous)
[26]	✓	✓				✓				✓	Multi-objective optimization (Pareto-based deployment)
Our Work	✓	✓	✓		✓		✓	✓			Multi-objective distributed optimization (all objectives jointly optimized)

## 3. Basic Concepts

This section aims to provide the fundamental concepts underlying the techniques employed in our study, providing a clear foundation for understanding the proposed approach prior to the implementation details. Understanding these concepts provides clarity on how each technique contributes to solving the edge-UAV deployment problem proposed in this paper.

### 3.1. Particle Swarm Optimization (PSO) Meta-Heuristic for UAV Deployment

PSO is a population-based meta-heuristic inspired by the social behavior of flocking birds or schooling fish. The optimization of UAV deployment based on the standard PSO meta-heuristic is modeled as a swarm of particles, where each particle represents a potential UAV deployment solution [23]. To search for the optimal solution, each particle updates its position and velocity in the solution space based on its own historical best position (pbest) and the global best position identified by the swarm (gbest) [27].

(1) $$\mathrm{pbest}\left(i,t\right)=\arg\min_{\begin{array}{c} k=1,\ldots ,t \end{array}}\left[f(P_{i}\left(k\right))\right],\;i\in \left\{1,2,\ldots ,N_{p}\right\}$$

(2) $$\mathrm{gbest}\left(t\right)=\arg\min_{k}\left[f(P_{i}\left(k\right))\right]$$

where *i* denotes the particle index, *f* represents the fitness function, *t* is the current iteration number, $N_{p}$ is the total number of particles, and *P* indicates the position of the particle. The position *P* and velocity *V* of the particles are updated by the following equations [27]:

(3) $$V_{i}\left(t+1\right)=\omega V_{i}\left(t\right)+c_{1}r_{1}\left(\mathrm{pbest}\left(i,t\right)-P_{i}\left(t\right)\right)+c_{2}r_{2}\left(\mathrm{gbest}\left(t\right)-P_{i}\left(t\right)\right)\;$$

(4) $$P_{i}\left(t+1\right)=P_{i}\left(t\right)+V_{i}\left(t+1\right)$$

where ω is the inertia weight used to balance global exploration and local exploitation; $r_{1}$ and $r_{2}$ are uniformly distributed random variables within the range [0,1]; and $c_{1}$ and $c_{2}$ are positive constants known as “acceleration coefficients”.

### 3.2. The Weighted-Sum Method

The Weighted-sum method is one of the most widely used scalarization techniques [5]. It transforms a MOP into a set of single-objective problems by assigning a specific weight to each objective (by varying the importance of objectives, we build a set of single-objective optimization subproblems), allowing them to be aggregated into a single scalar value.

(5) $$F_{\mathrm{fitness}}=\alpha \cdot f_{1}+\beta \cdot f_{2}+\ldots +\gamma \cdot f_{n}$$

where $f_{1}$, $f_{2}$, …, and $f_{n}$ represent the objectives to be optimized, and α,β,γ are weighting coefficients that control the importance given to each objective.

#### Normalization of Terms

The objective values in our paper (minimizing latency, maximizing coverage, and minimizing energy consumption) have different units and magnitudes. To ensure that all objectives are comparable and consistently directed toward minimization, we follow the standard optimization practice of reversing the sign of maximization objectives, as described by [28]: Any maximization problem can be transformed into a minimization problem by replacing the objective f(x) with −f(x). Thus, the coverage metric is converted to a minimization objective simply by using the following:

(6) $$Coverage_{norm}=-Coverage$$

### 3.3. The Epsilon-Constrained Method

The Epsilon constraint method is a mathematical programming technique that transforms a Multi-objective Optimization Problem (MOP) into a series of single-objective constrained problems. As a scalarization approach, it generates one Pareto front point at a time by optimizing one objective while treating the others as constraints [29]. The method typically assumes that all objective functions are to be minimized, as shown below:

(7) $$\left\{\begin{array}{c} \begin{array}{ccccc} \min\;f_{l}\left(x\right)\\ \mathrm{subject}\;\mathrm{to}\; & f_{j}\left(x\right)\leq \varepsilon_{j},\;\forall j=1,2,\ldots ,m,\;j\neq l\; & \; & & x\in S \end{array} \end{array}\right.$$

where l∈{1,2,…,m} and *S* define the feasible region, which is defined by a set of equality and/or inequality constraints. The vector of upper bounds, $\varepsilon =(\varepsilon_{1},\varepsilon_{2},\ldots ,\varepsilon_{m})$, specifies the maximum value for each objective function.

### 3.4. The Pareto Front in a Multi-Objective Optimization Problem (MOP)

**Figure 1:**
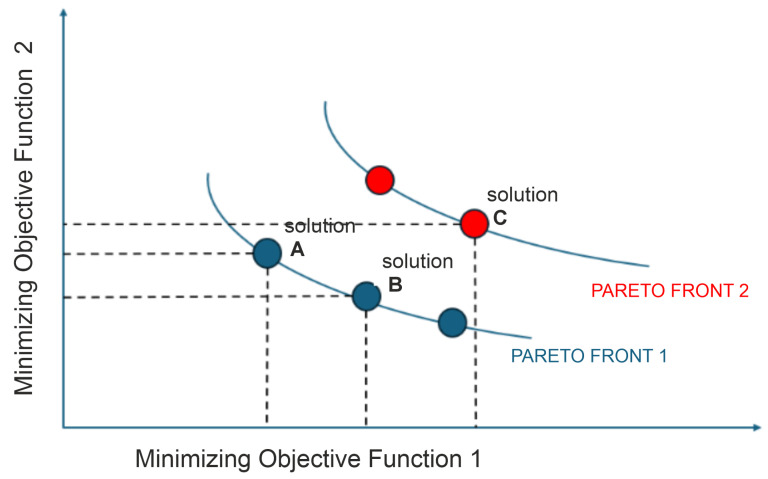
The set of objective space solutions for objective functions f1 and f2 along with A, B, and C solutions.

Multi-objective optimization is the process of simultaneously optimizing two or more objectives subject to certain constraints [30]. The process involves the simultaneous optimization of multiple objective functions that may conflict with each other, all within specified criteria. Due to the conflicting nature of these objectives and their associated constraints, determining an optimal set of solutions presents a significant challenge. A solution is deemed Pareto optimal when there is no other feasible solution that can enhance one objective without compromising at least one of the other objectives [31]. A MOP is formulated as follows in the study [30]:

(8) $$\min_{x\in X}f\left(x\right)=\left(f_{1}\left(x\right),f_{2}\left(x\right),\ldots ,f_{p}\left(x\right)\right)$$

where $X\subset R^{n}$ is a feasible nonempty set, and *f* is a vector-valued function composed of *p* (p≥2) real-valued functions. In Figure 1, all points in the dataset represent feasible solutions obtained for objective functions 1 and 2. When considering points A, B, and C with the objective of minimizing both objectives, the following observations can be made:

- Point A dominates point B in objective 1 and point C in both objectives.
- Point B dominates point A in objective 2 and also dominates point C in both objectives.
- Point C is dominated by both points A and B in both objectives.

In this scenario, points A and B are non-dominated by each other and by any other solution, which categorizes them as belonging to the same Pareto front (PF1). On the other hand, point C is dominated by both A and B, placing it in the subsequent Pareto front (PF2).

To allocate all individuals to distinct Pareto fronts, the process starts by identifying all non-dominated solutions (PF1) within the complete set of solutions *X*. The remaining set X−PF1 is then analyzed to determine the subsequent set of non-dominated solutions as PF2. This process is repeated iteratively until all solutions have been assigned to a Pareto front [31].

## 4. System Model and Problem Formulation

**Figure 2:**
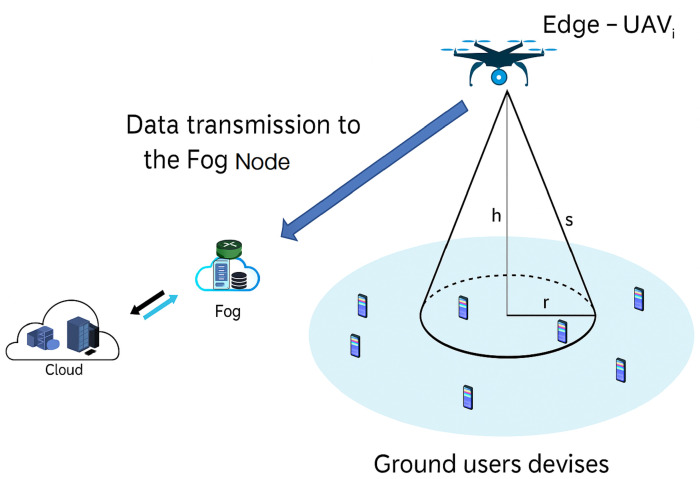
Communication architecture.

Figure 2 illustrates the communication architecture used for data reception and transmission. We consider a set N = {1, 2, 3, …, n} of UAVs equipped with edge computing capabilities, providing data collection services to a set M = {1, 2, 3, …, m} of GUs, distributed over a 500 m × 500 m geographical area. Vector S*i* = {$S_{i1}$, $S_{i2}$, $S_{i3}$, …, $S_{ij}$} represents the size of the data received from the GUs by edge-UAV*_i_*. We propose the following scenario:

- Edge-UAVs fly over the area of interest, providing connectivity to GU devices.
- GU devices upload their data, such as text messages, GPS positions, videos, and audio files.
- Each edge-UAV waits until it has received all assigned data and then retransmits it to a fog node for further processing.
- The new optimized position of each edge-UAV is computed using the PSO algorithm, which aims to optimize three objectives: maximizing the total non-overlapping coverage area, minimizing latency, and minimizing energy consumption.

### 4.1. Latency Model

In wireless communication, path loss is always present [23]. In our model, the overall path loss occurs in two stages: from GUs to edge-UAV*_i_* and from edge-UAV*_i_* to the fog node. The proposed system model is constructed based on the approach described in [27]. Each edge-UAV*_i_* receives data from several GUs and then forwards them to a shared fog node. For the reception stage, from ground users (GUs) to edge-UAVs, the communication model incorporates realistic path loss including both line-of-sight (LoS) and non-line-of-sight (NLoS) conditions probabilistically, as is consistent with standards such as 3GPP. Importantly, fading and shadowing effects are integrated to capture small- and large-scale channel variations. The achievable transmission rate is determined based on the Signal-to-Interference-plus-Noise Ratio (SINR), which includes interference arising from concurrent transmissions of other edge-UAVs. Similarly, for the transmission stage from edge-UAVs to the fog node, the same comprehensive path loss model is adopted, incorporating fading, shadowing, and interference effects akin to those considered in the GUs-to-UAV links. This unified approach enhances the accuracy and consistency of channel modeling across both communication stages. Consequently, the total delay experienced by an edge-UAV comprises these two principal components:

- Reception latency (Trecv): the time required for edge-UAV*_i_* to receive data from its associated GUs.
- Offloading latency (Toffload): the time needed to transmit the received data from edge-UAV*_i_* to the fog node.

In our latency model, we assume that edge-UAVs are equipped with a single radio interface, and data reception from GUs is performed sequentially. This serial reception mechanism reflects the practical limitations of UAV hardware and aligns with the rapid deployment objectives as access point UAVs, where implementing complex parallel reception schemes such as OFDMA may be constrained by size, power, and real-time processing capabilities. While parallel reception is common in modern UAV communication systems, and can help at reducing latency by supporting simultaneous multi-user transmissions, our adoption of sequential reception allows for analytical tractability and a clear characterization of worst-case latency accumulation. In particular, serial reception ensures that the total reception latency $T_{\mathrm{recv}}$ is determined by the slowest user transmission, as is consistent with Equation (13). Therefore, the total latency for edge-UAV*_i_* is given by Equation (9).

(9) $${\mathrm{Total}\_\mathrm{Latency}}_{i}=T_{\mathrm{recv},i}+T_{\mathrm{offload},i}$$

The power received on edge-UAV*_i_* from GU*_j_* is given by

(10) $$P_{r}\left(j\right)=\frac{P_{t}\;G\;\lambda^{2}\cdot F\cdot S}{{\left(4\pi d_{j,i}\right)}^{2}\cdot L_{\mathrm{LoS}/\mathrm{NLoS}}\left(d_{j,i}\right)}$$

where $P_{r}\left(j\right)$ is the received power at edge-UAV*i* from GU*_j_*, and $P_{t}$ is the transmission power of GU*_j_*. *G* denotes the total antenna gain (in dB), which includes the gains of both the transmitting antenna (Tx) in the ground device and the receiving antenna (Rx) in edge-UAV*_i_*. λ is the wavelength of the transmitted signal, given by $\lambda =c/f_{c}$, where *c* is the speed of light, and $f_{c}$ is the carrier frequency. F models the small-scale fading effect, commonly assumed Rayleigh-distributed representing multipath fluctuations, while S accounts for large-scale shadowing, modeled as a log-normal random variable reflecting slow environment changes such as obstacles. $d_{j,i}$ is the distance between edge-UAV*_i_* and GU*_j_*, and $L_{\mathrm{LoS}/\mathrm{NLoS}}\left(d_{j,i}\right)$ represents the path loss under LoS or NLoS conditions, respectively.

#### 4.1.1. Reception Latency ($T_{\mathrm{recv}}$)

As already defined, reception latency represents the time required for edge-UAV*_i_* to receive data from its associated GUs. The SINR at edge-UAV*i* for user *j* is calculated using the following equation:

(11) $${\mathrm{SINR}}_{j,i}=\frac{P_{r}\left(j\right)}{I+B\cdot N_{0}}$$

where $P_{r}\left(j\right)$ is the received signal power from ${\mathrm{GU}}_{j}$. $N_{0}$ is the noise power spectral density, I is the aggregate interference power from other concurrent transmission, and B is the system bandwidth. Based on this, the maximum transmission data rate ($T_{j}{,}_{i}$) from GU*_j_* to edge-UAV*_i_* can be expressed by the following equation:

(12) $$T_{j,i}=B\log_{2}\;\left(1+{\mathrm{SINR}}_{j,i}\right)$$

The reception latency is determined by Equation (13), which clearly indicates that the system performance depends on the slowest data reception among all GUs.

(13) $$T_{\mathrm{recv},i}=\max_{j}\left(\frac{S_{j,i}}{T_{j,i}}\right)$$

In this context, $S_{j,i}$ denotes the size of the data received from GU*_j_* by edge-UAV*_i_*, and $T_{j,i}$ represents the transmission data rate between GU*_j_* and edge-UAV*_i_*.

#### 4.1.2. Offloading Latency ($T_{\mathrm{offload}}$)

Once edge-UAV*_i_* has received all data, it offloads them to the fog node. The transmission rate from edge-UAV*_i_* to the fog node is given by Equation (16).

(14) $$P_{r}\left(i\right)=\frac{P_{t}\;G\;\lambda^{2}\cdot F\cdot S}{{\left(4\pi d_{i,\mathrm{FogNode}}\right)}^{2}\cdot L_{\mathrm{LoS}/\mathrm{NLoS}}\left(d_{i,\mathrm{FogNode}}\right)}$$

This equation represents the power received $P_{r}\left(i\right)$ at the receiver fog node from the transmitter edge-UAV*_i_* while considering the path loss due to the environment under line-of-sight (LoS) or non-line-of-sight (NLoS) conditions. Here, *G* represents the combined antenna gain of the transmitter and receiver, λ is the wavelength of the transmitted signal (in meters), and $d_{i,\mathrm{FogNode}}$ represents the distance between edge-UAV*_i_* and the fog node (in meters). F and S represent small-scale Rayleigh fading and large-scale log-normal shadowing random variables, respectively. The corresponding SINR at the fog node is expressed as

(15) $${\mathrm{SINR}}_{\mathrm{fogNode}}=\frac{P_{r}\left(i\right)}{I_{\mathrm{fogNode}}+N_{0}B}$$

where $P_{r}\left(i\right)$ is the received power from edge-UAV *i* at the fog node, $I_{\mathrm{fog}}$ denotes the interference power at the fog node from other UAVs, $N_{0}$ is the noise power spectral density, and *B* is the bandwidth. Now, the offloading data rate is given by the following equation:

(16) $$T_{i,\mathrm{FogeNode}}=B\cdot \log_{2}\left(1+{\mathrm{SINR}}_{i,\mathrm{FogeNode}}\right)$$

while the offloading latency is given by

(17) $$T_{\mathrm{offload},i}=\frac{\sum_{j}S_{j,i}}{T_{i,\mathrm{FogNode}}}$$

#### 4.1.3. Total Latency

The total latency is calculated by Equation (18):

(18) $${\mathrm{Total}\_\mathrm{Latency}}_{i}=\max\left(\frac{S_{j,i}}{T_{j,i}}\right)+\frac{\sum_{j}S_{j,i}}{T_{i,\mathrm{FogNode}}}$$

Algorithm 1 presents the pseudo-code for calculating the Total_Latency*_i_* associated with edge-UAV*_i_*. The algorithm is structured into three main parts; the reception latency calculation part measures how long it takes for edge-UAV*_i_* to receive data from all connected GUs, where the maximum latency (the slowest) among all users determine the completion time. The second part is the calculation of offloading latency, where the time required for edge-UAV*_i_* to offload all received data to a nearby fog node. The third part is the final aggregation part, where the total latency is the sum of the two latencies.
**Algorithm 1** Latency calculation.**Require:** UAV position UAVpos, user positions and data sizes Users, fog node position FogNode, transmit power $P_{\mathrm{trans}}$, noise spectral density $N_{0}$, bandwidth *B*, interfering UAVs Interferers**Ensure:** 
Total latency latency   1:Set small constant $\epsilon \leftarrow 10^{-9}$   2:Initialize max reception latency $T_{\mathrm{recv}}\leftarrow 0$   3:**if** Users is empty **then**   4:    latency←∞   5:    **return**   6:Set constants: speed of light *c*, carrier frequency $f_{c}$, wavelength $\lambda \leftarrow \frac{c}{f_{c}}$   7:Antenna gains $G_{\mathrm{recv}}$, $G_{\mathrm{offload}}$   8:Shadowing standard deviation $\sigma_{\mathrm{shadow}}$, fading mean *a*   9:Path loss constants $L_{\mathrm{LoS}}$, $L_{\mathrm{NLoS}}$ 10:**for** each user *j* **do** 11:     Calculate horizontal distance and elevation angle to UAV 12:     Compute LoS probability $P_{\mathrm{LoS}}$ based on elevation 13:     Draw random number, select extra loss $L_{\mathrm{extra}}$ (LoS or NLoS) 14:     Draw Rayleigh fading $f_{j}$ and shadowing $s_{j}$ (log-normal) 15:     Calculate path loss $PL_{j}$ with $L_{\mathrm{extra}}$ 16:     Compute received power $P_{r,j}=\frac{P_{\mathrm{trans}}G_{\mathrm{recv}}f_{j}}{PL_{j}s_{j}}$ 17:     Calculate interference power $I_{j}$ as sum over interferers:$$I_{j}=\sum_{m}\frac{P_{t,m}G_{\mathrm{recv}}}{PL_{j,m}}$$ 18:     Calculate ${\mathrm{SINR}}_{j}=\frac{P_{r,j}}{I_{j}+N_{0}B}$ 19:     Compute data rate $r_{j}=B\log_{2}\left(1+\max\left({\mathrm{SINR}}_{j},0\right)\right)$ 20:     Calculate reception time $T_{j}=\frac{S_{j}}{r_{j}}$ 21:     $T_{\mathrm{recv}}\leftarrow \max\left(T_{\mathrm{recv}},T_{j}\right)$ 22:Calculate horizontal distance and elevation angle to fog node 23:Compute LoS probability $P_{\mathrm{LoS},\mathrm{fog}}$ 24:Draw random, select loss $L_{\mathrm{extra},\mathrm{fog}}$ 25:Draw Rayleigh fading $f_{\mathrm{fog}}$ and shadowing $s_{\mathrm{fog}}$ 26:Calculate path loss $PL_{\mathrm{fog}}$ and received power $P_{r,\mathrm{fog}}$ 27:Calculate fog interference power $I_{\mathrm{fog}}=\sum_{k}\frac{P_{t,k}G_{\mathrm{offload}}}{PL_{\mathrm{fog},k}}$ 28:Calculate ${\mathrm{SINR}}_{\mathrm{fog}}=\frac{P_{r,\mathrm{fog}}}{I_{\mathrm{fog}}+N_{0}B}$ 29:Compute offload rate $r_{\mathrm{fog}}=B\log_{2}\left(1+\max\left({\mathrm{SINR}}_{\mathrm{fog}},0\right)\right)$ 30:Calculate offloading time $T_{\mathrm{offload}}=\frac{\sum_{j}S_{j}}{r_{\mathrm{fog}}}$ 31:**return** $\mathrm{latency}=T_{\mathrm{recv}}+T_{\mathrm{offload}}$

### 4.2. Energy Consumption Model

In this subsection, we introduce our energy consumption model for edge-UAVs, taking into consideration four main operations: communication, computation, hovering, and traveling. In general, the total energy consumption is calculated by adding the energy expended during data reception and transmission, local processing (PSO computation), hovering, and movement from a position to the new position.

#### 4.2.1. Energy Consumption for Data Reception and Transmission

The energy consumed during data reception and transmission depends on the transmission power of the communication system and the duration of these activities. Each edge-UAV handles communication in two phases: it receives data from ground devices during the first phase and then transmits the collected data to the fog node during the second phase, as described in [32]. We assume that each GU transmits *D* units of data to edge-UAV*_i_*, and the time required to complete this transmission is given by T=D/TR, where TR is the data rate. Since the energy consumed by edge-UAV*_i_* during reception is negligible (ignoring reception energy is a common and justified simplification in typical UAV data collection modeling, because the transmission workload is handled mainly by GUs), it can be ignored. Therefore, this assumption is supported by prior work such as [33], which explicitly neglects UAV communication energy including reception due to its relatively small magnitude compared to propulsion energy. The energy consumption of edge-UAVs is primarily considered during the data transmission phase to the fog node (data offloading) and can be modeled as in [32]:

(19) $$E_{\mathrm{trans}}=P_{\mathrm{trans}}\cdot \frac{D_{i}}{TR_{i}}$$

where $P_{\mathrm{trans}}$ is a constant power consumption of the communication module given by 0.5 watts in the simulation (assumed for a quadrotor UAV around 2.5–3 kg),  $D_{i}$ is the amount of data offloaded by edge-UAV*_i_* to the fog node, and $TR_{i}$ is the transmission rate between edge-UAV*_i_* and the fog node.

#### 4.2.2. Computational Energy

The energy required to execute the PSO algorithm to determine the next position is governed by the computational load and the power consumption of the onboard processor.

(20) $$E_{\mathrm{comp}}=P_{\mathrm{proc}}\times T_{\mathrm{comp}}$$

In Equation (20), $P_{\mathrm{proc}}$ represents the processor power consumption (in watts), and $T_{\mathrm{comp}}$ represents the time taken by each edge-UAV to run its own PSO runtime measured via tic/toc (in seconds).

#### 4.2.3. Traveling Energy

The traveling energy includes only the time required to move from the current position to the newly calculated position, as defined in Equation (21):

(21) $$E_{\mathrm{trav}}=P_{\mathrm{trav}}\times T_{\mathrm{trav}}$$

#### 4.2.4. Hovering Energy

In the context of UAV-assisted edge computing, hovering time refers to the total duration during which an Unmanned Aerial Vehicle (UAV) remains stationary in the air over a designated area to perform data-related operations. In our energy model, this includes the time spent with the edge-UAV for data reception from GUs, data transmission (offloading) to a fog node, and local computation. Formally, the hovering time $T_{\mathrm{hover},i}$ for edge-UAV*_i_* can be expressed as the sum of the communication and computation durations:

$$T_{\mathrm{hover},i}=T_{\mathrm{recv},i}+t_{\mathrm{offload},i}+T_{\mathrm{comp},i}$$

The hovering energy for edge-UAV*i* is defined by Equation (22).

(22) $$E_{\mathrm{hov}}=P_{\mathrm{hov}}\times T_{\mathrm{hov}}$$

where $P_{\mathrm{hov}}$ represents the power required for hovering (in watts), and $T_{\mathrm{hov}}$ is the hovering time (in seconds).

#### 4.2.5. Total Energy Consumption

By adding all components, the total energy consumption for the mission is given by

(23) $$E_{\mathrm{total}}=E_{\mathrm{hov}}+E_{\mathrm{trans}}+E_{\mathrm{comp}}+E_{\mathrm{trav}}$$

In our context, each term corresponds to the energy consumed during hovering, communication, computation, and movement phases, respectively. Algorithm 2 presents the pseudo-code used to calculate the energy consumption of each edge-UAV.
**Algorithm 2** Energy consumption calculation.**Require:** 
Previous UAV position prevPos, new UAV position newPos, Users Users, Fog node position FogNode, transmit power $P_{\mathrm{trans}}$, processing power $P_{\mathrm{proc}}$, traveling power $P_{\mathrm{travel}}$, hovering power $P_{\mathrm{hover}}$, noise spectral density $N_{0}$, bandwidth *B*, computation time $T_{\mathrm{comp}}$, interfering UAVs Interferers**Ensure:** 
Total energy consumption *E*  1:Set small constant $\epsilon \leftarrow 10^{-9}$  2:Calculate travel distance $d_{\mathrm{travel}}\leftarrow ∥\mathrm{newPos}-\mathrm{prevPos}∥$  3:Compute travel time $t_{\mathrm{travel}}\leftarrow \frac{d_{\mathrm{travel}}}{\max(\mathrm{speed},\epsilon )}$  4:Travel energy $E_{\mathrm{travel}}\leftarrow P_{\mathrm{travel}}\times t_{\mathrm{travel}}$  5:**if** Users is empty **then**  6:    Hover time $t_{\mathrm{hover}}\leftarrow T_{\mathrm{comp}}$  7:    Hover energy $E_{\mathrm{hover}}\leftarrow P_{\mathrm{hover}}\times t_{\mathrm{hover}}$  8:    Processing energy $E_{\mathrm{proc}}\leftarrow P_{\mathrm{proc}}\times T_{\mathrm{comp}}$  9:    Transmission energy $E_{\mathrm{trans}}\leftarrow 0$10:    Total energy $E\leftarrow E_{\mathrm{travel}}+E_{\mathrm{hover}}+E_{\mathrm{proc}}+E_{\mathrm{trans}}$11:    **return** *E*12:Initialize received powers from users to UAV: $PR_{\mathrm{users}\to \mathrm{UAV}}\leftarrow 0$13:**for** each user *j* **do**14:    Calculate distance $d_{j}$ between newPos and user *j*15:    Compute elevation and LoS probability $P_{\mathrm{LoS}}$16:    Randomly select loss factor $L_{\mathrm{extra}}$ for LoS/NLoS17:    Calculate free-space path loss $FSPL_{j}$18:    Draw fading $f_{j}$ and shadowing $s_{j}$19:    Received power:$$PR_{\mathrm{users}\to \mathrm{UAV}}\left(j\right)=\frac{P_{\mathrm{trans}}\times G_{\mathrm{recv}}\times f_{j}}{FSPL_{j}\times s_{j}}$$20:Calculate reception latency $T_{\mathrm{recv}}$ as the maximum user data transmission times based on SINR including interference21:Calculate fog offloading distance $d_{\mathrm{fog}}$ and LoS probability $P_{\mathrm{LoS},\mathrm{fog}}$24:Draw fading $f_{\mathrm{fog}}$ and shadowing $s_{\mathrm{fog}}$23:Calculate fog path loss and received power:$$P_{r,\mathrm{fog}}=\frac{P_{\mathrm{trans}}\times G_{\mathrm{off}}\times f_{\mathrm{fog}}}{PL_{\mathrm{fog}}\times s_{\mathrm{fog}}}$$24:Calculate interference at fog node $I_{\mathrm{fog}}$ from interferers similarly including fading and shadowing25:Compute SINR and offloading rate $r_{\mathrm{fog}}$26:Aggregate user data size $D_{\mathrm{total}}$, offload time:$$T_{\mathrm{offload}}=\frac{D_{\mathrm{total}}}{r_{\mathrm{fog}}}$$27:Calculate transmission energy:$$E_{\mathrm{trans}}=P_{\mathrm{trans}}\times \left(T_{\mathrm{recv}}+T_{\mathrm{offload}}\right)$$28:Processing energy:$$E_{\mathrm{proc}}=P_{\mathrm{proc}}\times T_{\mathrm{comp}}$$29:Hover time and energy:$$t_{\mathrm{hover}}=T_{\mathrm{recv}}+T_{\mathrm{offload}}+T_{\mathrm{comp}}$$$$E_{\mathrm{hover}}=P_{\mathrm{hover}}\times t_{\mathrm{hover}}$$30:Total energy consumption:$$E=E_{\mathrm{travel}}+E_{\mathrm{hover}}+E_{\mathrm{proc}}+E_{\mathrm{trans}}$$31:**return** *E*

### 4.3. Coverage Model

The coverage model evaluates how effectively edge-UAVs cover the target area while minimizing overlapping coverage. The objective is to maximize the total non-overlapping coverage area, thereby ensuring efficient spatial utilization and reducing redundancy.

In our deployment scenario, the coverage area is determined by the union of the coverage disks provided by all edge-UAVs. When multiple edge-UAVs operate in close proximity, their coverage regions can overlap, which can lead to an overestimation of the total covered area if not treated carefully. To ensure accurate estimation, we adopt the *inclusion–exclusion* principle that systematically accounts for pairwise, triple, and higher-order overlaps. Let $C_{i}$ denote the coverage area of edge-UAV*_i_*, defined as a disk of radius $R_{i}$ centered at the edge-UAV’s ground projection. The total coverage area $A_{\mathrm{cov}}$ of *N* edge-UAVs is given by the following union:

(24) $$A_{\mathrm{cov}}=|\overset{N}{\underset{i=1}{⋃}}C_{i}|.$$

Applying the inclusion–exclusion formula, we expand the union as follows:

(25) $$\begin{array}{cc} A_{cov}=\; & \sum_{i=1}^{N}|C_{i}|-\sum_{1\leq i<j\leq N}\left|C_{i}\cap C_{j}\right|\\ \; & +\sum_{1\leq i<j<k\leq N}\left|C_{i}\cap C_{j}\cap C_{k}\right|-\cdots \\ \; & +{\left(-1\right)}^{m+1}\sum_{1\leq i_{1}<\cdots <i_{m}\leq N}|\overset{m}{\underset{l=1}{⋂}}C_{i_{l}}| \end{array}$$

Here, we have the following:

- $|C_{i}|=\pi R_{i}^{2}$ represents the area covered by edge-UAV *i*;
- $|C_{i}\cap C_{j}|$ is the intersection area between edge-UAV *i* and edge-UAV *j*;
- Higher-order intersections $|C_{i}\cap C_{j}\cap C_{k}|$, and others, capture regions simultaneously covered by three or more edge-UAVs.

Algorithm 3 illustrates how to compute the total non-overlapping coverage area of multiple edge-UAVs. In practice, we implement the inclusion–exclusion principle up to triple overlaps (pairwise and triple intersections), which provides an accurate and computationally tractable estimate of the non-overlapping coverage.
**Algorithm 3** Non-overlapping coverage calculation.**Require:** UAV positions $UAV=\{u_{1},\ldots ,u_{N}\}$, coverage radius *R***Ensure:** Total non-overlapping coverage area *C* **if** N=0 **then**       C←0       **return**$total\_area\leftarrow N\cdot \pi R^{2}$pairwise_overlap←0**for** i←1 to N−1 **do**    **for** j←i+1 to *N* **do**        $d\leftarrow ∥u_{i}-u_{j}∥$        **if** d<2R **then**           pairwise_overlap←pairwise_overlap+CIRCLEOVERLAP(R,d)triple_overlap←0**for** i←1 to N−2 **do**    **for** j←i+1 to N−1 **do**        **for** k←j+1 to *N* **do**           $triple\_overlap\leftarrow triple\_overlap+\mathrm{TRIPLECIR}C\mathrm{IRCLE}O\mathrm{VERLAP}(u_{i},u_{j},u_{k},R)$C←total_area−pairwise_overlap+triple_overlap**return** *C*

### 4.4. Integration of PSO-Based Methods with Our Proposed Models

In this section, the integration of our suggested Pareto-PSO for edge-UAV deployment is explained by contrasting it with two alternative PSO methods: Epsilon-constrained PSO and Weighted-sum PSO. These approaches vary in their strategies for computing the fitness function when dealing with numerous objectives. The are defined as follows:

- Epsilon-constrained PSO: In this implementation of the PSO meta-heuristic, we use a rotating primary objective while treating the other two objectives as constraints. We are interested in the first case of considering the maximization of the coverage area as the main objective to optimize and the minimization of both latency and energy consumption as constraints. Then, we switch to the other objectives by role. By doing this, we generate a series of single-objective optimization subproblems. In the basic concept of the Epsilon-constrained PSO method, we can sweep the epsilon value for each primary objective to increase the Pareto front of optimal solutions. In this study, a single ϵ value is used for each constraint to maintain a consistent computational framework across compared methods. The goal is to evaluate convergence and relative performance rather than to construct the complete Pareto front. The selected ε thresholds are empirically determined to reflect realistic and feasible operating conditions. A penalty is used to prevent the optimizer from favoring solutions that seem good in other objectives but violate practical constraints. Then, the PSO meta-heuristic is applied to the obtained fitness functions. Algorithm 4 illustrates how the Epsilon-constrained PSO handles and evaluates the fitness function by rotating the primary objectives.

**Algorithm 4** Fitness function definition for Epsilon-constrained PSO.**Require:** New position $P_{\mathrm{new}}$, previous position $P_{\mathrm{prev}}$, Users, FogNode, radius *R*, thresholds $\epsilon_{\mathrm{energy}},\epsilon_{\mathrm{latency}},\epsilon_{\mathrm{coverage}}$, powers $P_{\mathrm{trans}},P_{\mathrm{proc}},P_{\mathrm{travel}},P_{\mathrm{hover}}$, $\beta_{0}$, $N_{0}$, *B*, primary_objective, compute time $T_{\mathrm{comp}}$.**Ensure:** fitness   1:$C\leftarrow \mathrm{COMPUTE}C\mathrm{OVERAGE}(P_{\mathrm{new}},R)$   2:$L\leftarrow \mathrm{COMPUTE}L\mathrm{ATENCY}(P_{\mathrm{new}},\mathrm{Users},\mathrm{FogNode},P_{\mathrm{trans}},\beta_{0},N_{0},B)$   3:$E\leftarrow \mathrm{COMPUTE}E\mathrm{NERGY}(P_{\mathrm{prev}},P_{\mathrm{new}},\mathrm{Users},\mathrm{FogNode},P_{\mathrm{trans}},P_{\mathrm{proc}},P_{\mathrm{travel}},P_{\mathrm{hover}},\;N_{0},B,\;T_{\mathrm{comp}})$   4:α←1000,β←1000,γ←1000   5:penalty←0   6:**if** primary_objective=coverage **then**   7:    **if** $E>\epsilon_{\mathrm{energy}}$ **then**   8:        $\mathrm{penalty}\leftarrow \mathrm{penalty}+\alpha \left(E-\epsilon_{\mathrm{energy}}\right)$   9:    **if** $L>\epsilon_{\mathrm{latency}}$ **then** 10:        $\mathrm{penalty}\leftarrow \mathrm{penalty}+\beta \left(L-\epsilon_{\mathrm{latency}}\right)$ 11:    fitness←−C+penalty 12:**else if** primary_objective=energy **then** 13:    **if** $L>\epsilon_{\mathrm{latency}}$ **then** 14:        $\mathrm{penalty}\leftarrow \mathrm{penalty}+\beta \left(L-\epsilon_{\mathrm{latency}}\right)$ 15:    **if** $C<\epsilon_{\mathrm{coverage}}$ **then** 16:        $\mathrm{penalty}\leftarrow \mathrm{penalty}+\gamma \left(\epsilon_{\mathrm{coverage}}-C\right)$ 17:    fitness←E+penalty 18:**else if** primary_objective=latency **then** 19:    **if** $E>\epsilon_{\mathrm{energy}}$ **then** 20:        $\mathrm{penalty}\leftarrow \mathrm{penalty}+\alpha \left(E-\epsilon_{\mathrm{energy}}\right)$ 21:    **if** $C<\epsilon_{\mathrm{coverage}}$ **then** 22:        $\mathrm{penalty}\leftarrow \mathrm{penalty}+\gamma \left(\epsilon_{\mathrm{coverage}}-C\right)$ 23:    fitness←L+penalty 24:**else** 25:    **error**: invalid primary objective 26:**return** fitness

- Weighted-sum PSO: In this implementation, an aggregate fitness is formed by assigning equal weights to the three objectives—maximizing coverage and minimizing latency and energy—thereby treating their importance as equal. In the Weighted-sum PSO implementation, equal weights (0.33, 0.33, 0.33) are assigned to the latency, energy, and coverage objectives to establish a balanced and unbiased baseline. The intention is to compare scalarization and Pareto-based strategies under uniform importance rather than to perform sensitivity analysis on weight tuning. The PSO metaheuristic is then applied to this scalarized objective. Algorithm 5 details how the Weighted-sum PSO evaluates a multi-objective formulation via scalarization.

**Algorithm 5** Fitness function definition for Weighted-sum PSO.
**Require:** Previous position $P_{\mathrm{prev}}$, new position $P_{\mathrm{new}}$, users Users, Fog node FogNode, transmit power $P_{\mathrm{trans}}$, processing power $P_{\mathrm{proc}}$, travel power $P_{\mathrm{travel}}$, hover power $P_{\mathrm{hover}}$, pathloss coefficient $\beta_{0}$, noise power spectral density $N_{0}$, bandwidth *B*, weights $W_{\mathrm{latency}},W_{\mathrm{coverage}},W_{\mathrm{energy}}$, coverage radius *R*, computation time $T_{\mathrm{comp}}$**Ensure:** Fitness value fitness  1:Compute energy consumption: $E\leftarrow \mathrm{computeEnergy}(P_{\mathrm{new}},P_{\mathrm{prev}},\mathrm{Users},\mathrm{FogNode},P_{\mathrm{trans}},$ $P_{\mathrm{proc}},P_{\mathrm{travel}},P_{\mathrm{hover}},N_{0},B,T_{\mathrm{comp}})$  2:Compute coverage: $C\leftarrow \mathrm{computeCoverage}(P_{\mathrm{new}},R)$  3:Compute latency: $L\leftarrow \mathrm{computeLatency}(P_{\mathrm{new}},\mathrm{Users},\mathrm{FogNode},P_{\mathrm{trans}},\beta_{0},N_{0},B)$  4:Calculate total fitness: $\mathrm{fitness}\leftarrow W_{\mathrm{latency}}\cdot L+W_{\mathrm{coverage}}\cdot \left(-C\right)+W_{\mathrm{energy}}\cdot E$  5:**return** fitness

- Pareto-PSO: In this implementation, the fitness is vector-valued and evaluated by Algorithm 6, combining latency, coverage, and energy so they are optimized simultaneously. Each edge-UAV independently executes a Pareto-PSO-based multi-objective routine on its dynamically assigned subset of GUs, yielding a decentralized, parallel set of optimizations rather than a single centralized swarm optimization. Each UAV produces a three-dimensional fitness vector (latency, coverage, energy) over its local service area.

**Algorithm 6** Fitness function definition for Pareto-PSO.
**Require:** Previous position $P_{\mathrm{prev}}$, new position $P_{\mathrm{new}}$, users Users, Fog node FogNode, coverage radius *R*, bandwidth *B*, pathloss coefficient $\beta_{0}$, noise power spectral density $N_{0}$, transmit power $P_{\mathrm{trans}}$, processing power $P_{\mathrm{proc}}$, travel power $P_{\mathrm{travel}}$, hover power $P_{\mathrm{hover}}$, computation time $T_{\mathrm{comp}}$**Ensure:** Fitness vector fitness=[L,C,E]  1:Compute latency: $L\leftarrow \mathrm{computeLatency}(P_{\mathrm{new}},\mathrm{Users},\mathrm{FogNode},P_{\mathrm{trans}},\beta_{0},N_{0},B)$  2:Compute coverage: $C\leftarrow -\mathrm{computeCoverage}(P_{\mathrm{new}},R)$  3:Compute energy consumption: $E\leftarrow \mathrm{computeEnergy}(P_{\mathrm{prev}},P_{\mathrm{new}},\mathrm{Users},\mathrm{FogNode},P_{\mathrm{trans}},$ $P_{\mathrm{proc}},P_{\mathrm{travel}},P_{\mathrm{hover}},N_{0},B,T_{\mathrm{comp}})$  4:Construct fitness vector: fitness←[L,C,E]  5:**return** fitness



Algorithm 7 implements a decentralized Pareto-based Particle Swarm Optimization (Pareto-PSO) for edge-UAV deployment. Each UAV executes an independent, locally informed PSO process in parallel with other UAVs. Although the architecture follows a decentralized and communication-light structure, it does not yet incorporate inter-UAV cooperation mechanisms typically seen in fully distributed systems. Therefore, the proposed framework is better characterized as a parallel decentralized optimization scheme rather than a fully cooperative distributed one. For each UAV, a local swarm of particles is initialized around its starting location, and each particle encodes a candidate 3D position. Fitness evaluation is performed using a vector of the three objectives. To guide particle movement, an archive of non-dominated solutions is maintained for each UAV. Leaders are selected via crowding distance: a leader is sampled from archive members with the largest crowding distance (least crowded region) to promote diversity; ties are broken at random, and boundary members retain maximal distance. Particle velocities and positions are then updated using classical cognitive and social terms, with fitness always treated as a vector, ensuring true Pareto dominance. Each UAV continues this local optimization until convergence, outputting its best position and associated fitness.

Crucially, our model incorporates user mobility by updating user positions at each iteration of the optimization. After UAVs reposition and users move, a dynamic user association is performed, where each user is attributed to the UAV providing the highest SINR, with explicit consideration of inter-UAV interference. This reassociation occurs iteratively in tandem with UAV position updates, ensuring users are consistently connected to the UAV offering the optimal link quality at each step. This adaptive mechanism helps mitigate the natural trade-off between maximizing coverage and minimizing latency by preventing users from being persistently served by UAVs with poor channel conditions or at long distances.

Once all UAVs converge, the selected positions are collected into bestPosMat. Total non-overlapping coverage is obtained by forming the union of coverage maps derived from bestPosMat, avoiding double counting. Global throughput is then computed once from bestPosMat under dynamic user association, where each user is attributed to the UAV providing the highest SINR while explicitly accounting for inter-UAV interference. In parallel, per-UAV archives are concatenated into a global swarm archive that approximates the system-level Pareto front with non-dominated solutions only and supports system-wide decision making. For each composition of UAV positions drawn from these archives, the aggregated non-overlapping coverage and total network latency are evaluated using dynamic user association and SINR-based reassignment, with interference explicitly considered. This post-processing step constructs a comprehensive set of multi-objective deployment scenarios, spanning a range of coverage and latency trade-offs.
**Algorithm 7** Decentralized Pareto-PSO for edge-UAV deployment.**Require:** 
Initial UAV positions ${\mathrm{UAVs}}_{init}$, user sets $\left\{U_{1},\ldots ,U_{N}\right\}$, fog node, bounds [lb,ub], number of particles *P*, maximum iterations *T***Ensure:** 
Optimized UAV positions, fitness vectors, non-overlapping coverage, global throughput, swarm archive  1:Initialize bestPosMat←0, bestFits←0, $A_{swarm}\leftarrow \emptyset$  2:Initialize global union coverage map $C_{union}\leftarrow 0$  3:**for** each UAV n=1…N **do**  4:    Initialize *P* particles around ${\mathrm{UAVs}}_{init}\left(n\right)$ with random velocities  5:    Initialize local archive $A_{n}\leftarrow \emptyset$  6:    **for** each particle *i* **do**  7:        Evaluate fitness $f_{i}=\left[\mathrm{latency},\mathrm{coverage},\mathrm{energy}\right]$  8:        Set personal best ${\mathrm{pbest}}_{i}\leftarrow f_{i}$  9:        $A_{n}\leftarrow \mathrm{updateArchive}\left(A_{n},f_{i}\right)$10:    **for** iter =1 to *T* **do**11:        **Update user positions via mobility model**:Users(:,1:3)←update_mobile_users(Users(:,1:3),userBounds,maxSpeed,ΔT)12:        **for** each particle *i* **do**13:           Select leader *l* from $A_{n}$ using crowding-distance–based selection14:           Update velocity:$$v_{i}\leftarrow wv_{i}+c_{1}r_{1}\cdot \left({\mathrm{pbest}}_{i}-x_{i}\right)+c_{2}r_{2}\cdot \left(\mathrm{leader}-x_{i}\right)$$15:           Update position:$$x_{i}\leftarrow x_{i}+v_{i}$$16:           Enforce bounds: $x_{i}\leftarrow \max\left(\min\left(x_{i},ub\right),lb\right)$17:           Recompute fitness $f_{i}=\left[\mathrm{latency},\mathrm{coverage},\mathrm{energy}\right]$ with updated Users18:           **if** $f_{i}$ dominates ${\mathrm{pbest}}_{i}$ **then**19:               ${\mathrm{pbest}}_{i}\leftarrow f_{i}$20:           $A_{n}\leftarrow \mathrm{updateArchive}\left(A_{n},f_{i}\right)$21:    Select balanced solution from $A_{n}$: ${\mathrm{pos}}_{n},f_{n}$22:    $\mathrm{bestPosMat}\left(n,:\right)\leftarrow {\mathrm{pos}}_{n}$, $\mathrm{bestFits}\left(n,:\right)\leftarrow f_{n}$23:    $C_{union}\leftarrow \max\left(C_{union},C\left({\mathrm{pos}}_{n}\right)\right)$                 ▹ Non-overlap coverage24:Compute union coverage: $Coverage=\sum C_{union}$25:Compute total throughput considering inter-UAV interference:26:**for** each user *u* **do**27:    Compute distances $d_{k,u}$ from each UAV *k* to user *u*28:    Compute gains $g_{k,u}=\beta_{0}/d_{k,u}^{2}$29:    Compute SINR for UAV *k*:$${\mathrm{SINR}}_{k,u}=\frac{P_{\mathrm{trans}}\cdot g_{k,u}}{N_{0}+\sum_{j\neq k}P_{\mathrm{trans}}\cdot g_{j,u}}$$30:    Assign user *u* to UAV $k*=\arg\max_{k}{\mathrm{SINR}}_{k,u}$31:    $TP\leftarrow TP+B\log_{2}\left(1+{\mathrm{SINR}}_{k*,u}\right)$32:Remove duplicate fitness entries from $A_{swarm}$33:**return** $\mathrm{bestPosMat},\;\mathrm{bestFits},\;Coverage,\;TP,\;A_{swarm}$

## 5. Performance Evaluation

To evaluate the performance of our proposed Pareto-PSO in comparison with other UAV deployment methods, we performed several simulations using MATLAB R2016a. The simulation parameters, selected after a series of experimental tests, are presented in Table 3. Our simulation parameters were chosen to reflect realistic UAV-assisted edge computing scenarios. Each UAV covered a circular area of radius R=200 m and operates in a 500 m-by-500 m square environment. The noise power spectral density was set to $n_{0}=3.16\times 10^{-14}$ watts, while the transmission bandwidth *B* was explored at four levels, from 200 kHz up to 40 MHz. We studied networks with 5 to 25 edge-UAVs (numUAVs) and 50 to 250 GUs (numUsers). Each simulation used 12 particles over 50 iterations to optimize deployment. The system constraints included a maximum energy consumption of $\varepsilon_{\mathrm{energy}}=4\times 10^{5}$ joules, a minimum coverage area of $\varepsilon_{\mathrm{coverage}}=4.71\times 10^{4}$ m^2^, and a latency threshold of $\varepsilon_{\mathrm{latency}}=0.8$ s. Realistic power values were used for transmission ($P_{\mathrm{trans}}=0.5$ W), processing ($P_{\mathrm{proc}}=20$ W), travel ($P_{\mathrm{travel}}=150$ W), and hovering ($P_{\mathrm{hover}}=200$ W). The baseline PSO and Weighted-sum PSO parameters reported in Table 3 ($W_{\mathrm{coverage}}=W_{\mathrm{latency}}=W_{\mathrm{energy}}=0.33$, inertia w=0.7, learning coefficients $c_{1}=c_{2}=1.5$) were chosen for two reasons: (1) they represent balanced trade-offs (equal weighting) suitable for rapid-deployment scenarios where no single objective should dominate initial decisions, and (2) the PSO coefficients follow widely accepted settings from the PSO literature that provide good convergence and maintain a balance between exploration and exploitation in swarm intelligence studies. The optimization framework utilizes a particle swarm algorithm with 12 particles and 50 iterations to maximize coverage, minimize latency, and optimize energy consumption while adhering to practical operational constraints. Our proposed Pareto-PSO for edge-UAV deployment was compared with two PSO-based methods—Weighted-sum PSO and Epsilon-constrained PSO—according to their achieved throughput, coverage area, and computational convergence time. This setup provides a clear and reproducible means to benchmark the impact of different multi-objective decision-making methods on the performance and efficiency of a UAV-based edge network.

The simulation parameters were chosen according to standard UAV communication models and previous works [1,2,3,4,5,6,7,8,9,10].

### Experimental Results

**Figure 3:**
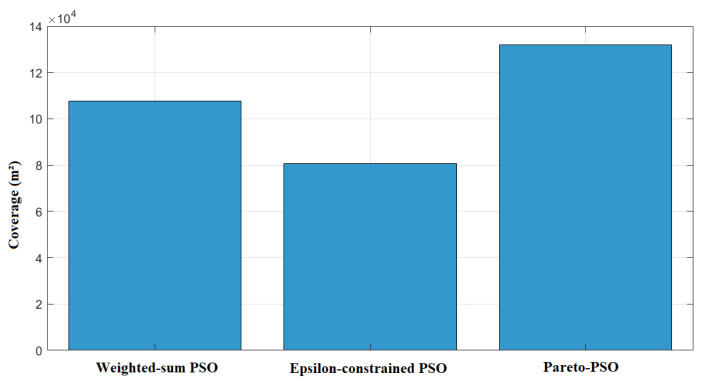
Coverage comparison of our proposed Pareto-PSO, Weighted-sum PSO, and Epsilon-constrained PSO.

Figure 3 contrasts the total non-overlapping coverage areas attained by the three optimization strategies. Pareto-PSO achieved the largest covered footprint at approximately 131,862 m^2^, indicating that the search successfully identified frontier configurations that extend spatial reach while remaining acceptable on the accompanying objectives. Weighted-sum PSO followed with about 107,511 m^2^, which is consistent with scalarization that can approximate a coverage-oriented solution when the weighting scheme affords sufficient emphasis to the area component yet still moderates extremes due to the composite objective. Epsilon-constrained PSO yielded the smallest coverage at roughly 80,637 m^2^ under the reported thresholds, reflecting the restrictive effect of feasibility limits: when stringent latency or energy bounds are enforced, the feasible region excludes some coverage-maximizing placements, thereby reducing aggregate served area.

**Figure 4:**
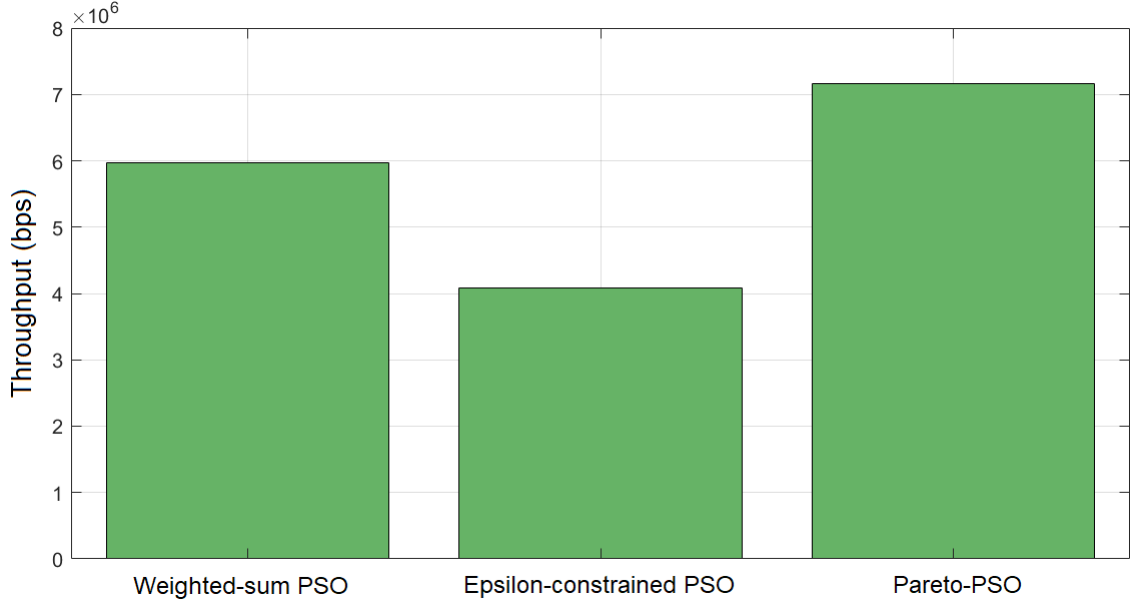
Throughput comparison of our proposed Pareto-PSO, Weighted-sum PSO, and Epsilon-constrained PSO.

In terms of throughput, Figure 4 reports the aggregate throughput achieved by each method. Pareto-PSO attained the highest rate, approximately 7.16 Mb/s (megabits per second), which is consistent with selecting a non-dominated solution located near the rate-optimal edge of the frontier. Weighted-sum PSO reached about 5.97 Mb/s, reflecting the expected attenuation caused by simultaneous optimization of competing objectives under fixed weights; unless the scalarization heavily privileges throughput, the best scalar solution typically falls short of the extreme. Epsilon-constrained PSO returned the lowest throughput, around 4.08 Mb/s, which accords with feasibility-driven optimization: tighter ϵ bounds on latency and/or energy reduce access to high-throughput configurations, yielding compliant but less rate-intensive deployments.

**Figure 5:**
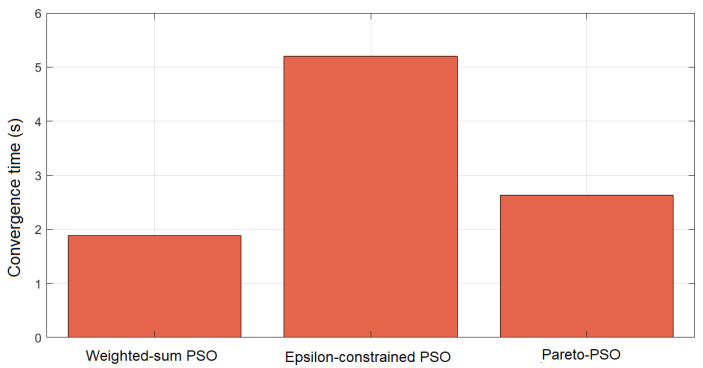
Comparison of convergence times for the proposed Pareto-based PSO, the Weighted-Sum PSO, and the Epsilon-Constrained PSO.

Figure 5 compares the convergence times across the three strategies for edge-UAV deployment. Weighted-sum PSO attained the shortest runtime at 1.769 s, underscoring the efficiency of scalarization. By reducing multiple objectives into a single scalar, the search becomes concentrated, decision overhead diminishes, and the swarm stabilizes rapidly. Pareto-PSO recorded an intermediate time of 2.600 s, which is consistent with the modest but necessary overhead of maintaining a non-dominated archive. Nevertheless, the archive’s elite solutions provide strong guidance, allowing the swarm to balance exploration and exploitation while converging within a reasonable time frame. The Epsilon-constrained PSO required the longest convergence time (5.134 s), reflecting the computational burden of feasibility screening and penalty enforcement. Each iteration must filter infeasible solutions and reorient the swarm toward admissible regions, increasing the evaluations needed before stabilization. While slower, this ensures strict compliance with system constraints.

The comparative results in Table 4 provide valuable insight into the behavior of Weighted-sum PSO (W_PSO), Epsilon-constrained PSO (Eps_PSO), and Pareto-PSO (P_PSO) under identical deployment conditions. The comparative results in these ten independent runs reveal clear performance distinctions among our proposed Pareto-PSO with the other PSO-based optimization methods for edge-UAV deployment. Regarding coverage, Pareto-PSO consistently achieved the highest values, ranking first in 10 out of 10 runs. This demonstrates its superiority in expanding non-overlapping UAV coverage and ensuring broader service areas. Weighted-sum PSO also performed competitively, with several runs showing near parity with Pareto-PSO (e.g., Runs 2 and 10), highlighting its stability in preserving spatial reach. In contrast, Epsilon-constrained PSO systematically yielded the lowest coverage values, which reflects its conservative optimization approach under strict feasibility constraints. The trade-off becomes more apparent when throughput is analyzed. Pareto-PSO clearly dominated this metric across 9 out of 10 runs, with values reaching up to 12.22Mb/s (Run 6), thereby confirming its effectiveness in directing UAVs toward high-demand regions and maximizing data transmission rates. Weighted-sum PSO maintained intermediate throughput levels, averaging around 6.3Mb/s, while Epsilon-constrained PSO lagged behind, rarely exceeding 5.6Mb/s. These observations confirm that Pareto-PSO prioritizes throughput maximization without sacrificing robustness in convergence. Overall, the evidence indicates that Pareto PSO offers the best compromise, combining high coverage, superior throughput, and moderate convergence times. Weighted-sum PSO remains attractive when speed is critical but sacrifices optimality, whereas Epsilon-constraint PSO provides strict feasibility but at the cost of both performance and efficiency. Finally, the convergence time reveals another layer of differentiation. Weighted-sum PSO proved to be the fastest across nearly all runs, with an average convergence time of ∼1.2 s, reflecting its computational simplicity and speed. Pareto-PSO, though slightly slower (∼1.7 s on average), yielded a difference that is not significant, and it still achieved a very satisfactory time and a balanced trade-off by exploring a wider solution space while still converging much faster than Epsilon-constrained PSO. The latter remained the slowest, requiring more than 3 s in all runs due to its constraint-handling overhead. These findings align with the theoretical expectation that Pareto-based optimization methods excel at balancing multiple objectives simultaneously, producing superior throughput and competitive coverage, while maintaining reasonable computational efficiency compared to more rigid approaches.

To assess robustness, we computed 95% confidence intervals (CIs) for the coverage, throughput, and convergence time across ten runs. Table 5 shows that Pareto-PSO clearly dominated by achieving both the highest coverage and the highest throughput (8.26 Mb/s, CI [7.27,9.24]), with consistent improvements over Weighted-sum and Epsilon-constrained PSO. Although Weighted-sum PSO converged faster, its coverage and throughput remained significantly lower. These results confirm that Pareto-PSO provides the most effective trade-off, ensuring superior coverage and reliable data-rate performance.

**Figure 6:**
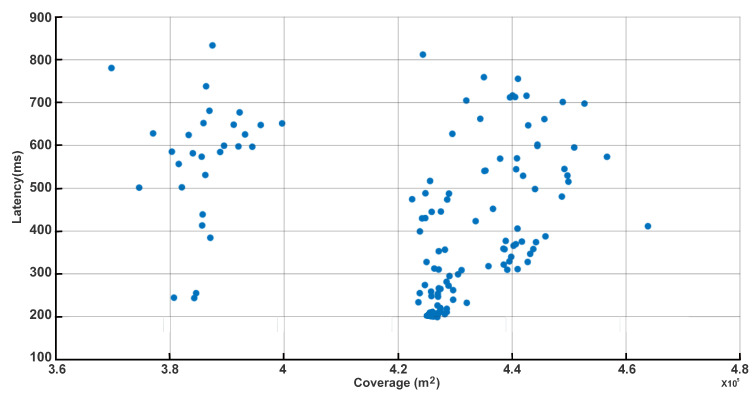
Pareto trade-off: coverage vs. latency.

The resulting Pareto front, shown in Figure 6, presents a robust and quantitative portrayal of the fundamental trade-off achieved by our decentralized edge-UAV swarm optimization. This front reveals a spectrum of optimal solutions in which maximizing coverage ($3.7\times 10^{5}\;m^{2}$ to $4.6\times 10^{5}\;m^{2}$) necessarily incurs higher latency values, sometimes reaching 800 ms, as UAVs must disperse to serve a wider area—thereby increasing communication distances and path delays. At the opposite end, deployments clustering UAVs nearer to user hotspots yield much lower latencies (as low as 200 ms) but must sacrifice total coverage. Crucially, the spread and diversity of the Pareto front vividly demonstrate that our algorithm effectively discovers a broad set of non-dominated trade-offs, capturing realistic system-level deployment scenarios aligned with practical needs and constraints. This diversity—distinct clusters and wide latency range—directly validates the flexibility and adaptability of our approach, letting system designers select deployments perfectly matched to specific operational priorities, whether for rapid response or maximal user reach. By presenting only non-dominated, genuinely optimal points, Figure 6 and Table 6 provide transparent, actionable guidance for UAV swarm planning and directly address the reviewer’s request for rigorous multi-objective analysis. The strength of the methodology is further highlighted by the absence of redundant or inferior configurations, ensuring every point on the front reflects a true performance compromise suitable for practical implementation.

**Figure 7:**
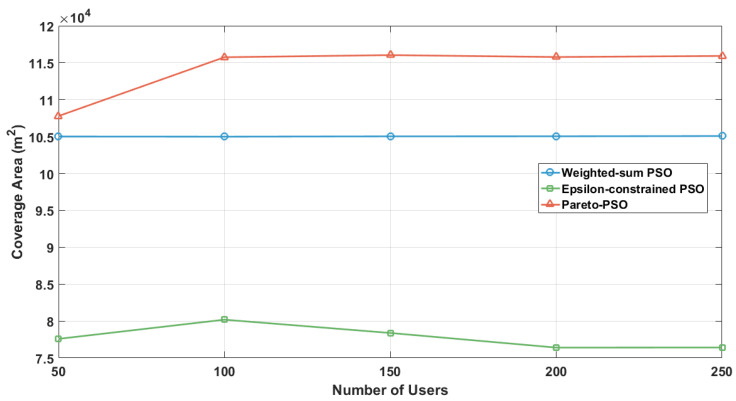
Users number influence on coverage area.

Figure 7 examines the evolution of non-overlapping coverage as the user density increased across the three multi-objective strategies: Weighted-sum PSO, Epsilon-constrained PSO, and Pareto-PSO. The trajectories reveal a consistent hierarchy: Pareto-PSO achieved the largest coverage envelope at all load levels, increasing from approximately $10.7\times 10^{4}\;m^{2}$ at 50 users to about $11.6\times 10^{4}\;m^{2}$ between 100 and 250 users. In contrast, the Weighted-sum PSO curve remained nearly flat at around $10.4\times 10^{4}\;m^{2}$, while the Epsilon-constrained PSO started lower (about $7.8\times 10^{4}\;m^{2}$ at 50 users), showed a temporary improvement at 100 users, but then declined to approximately $7.6\times 10^{4}\;m^{2}$ by 200–250 users. Overall, the results suggest that Pareto-PSO delivers the most robust coverage under varying demand, Weighted-sum PSO provides stable but less adaptive performance, and Epsilon-constrained PSO requires density-aware tuning of its feasibility schedule to mitigate coverage erosion as user density grows.

**Figure 8:**
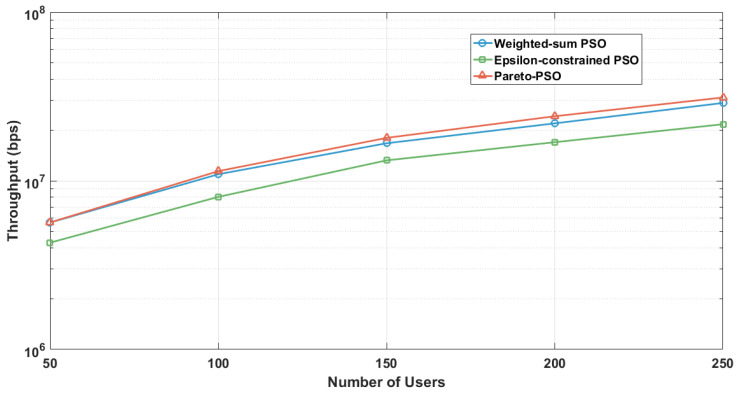
Users number influence on throughput.

Figure 8 presents the aggregate throughput as a function of user density across the three optimization strategies under the edge-UAV deployment scenario for data collection. Pareto-PSO consistently defined the upper performance envelope, increasing from approximately 5–6 Mb/s at 50 users to about 35–40 Mb/s at 250 users. Weighted-sum PSO closely paralleled this trend but remained marginally lower at all densities (e.g., 11–12 Mb/s versus 12–13 Mb/s at 100 users and roughly 30–35 Mb/s versus 35–40 Mb/s at 250 users). In contrast, Epsilon-constrained PSO exhibited steady improvement yet remained consistently below the other methods (e.g., 7–8 Mb/s at 100 users and 20–25 Mb/s at 250 users), reflecting the restrictive influence of feasibility enforcement at higher loads. Overall, these findings underscore that Pareto-PSO’s ability to preserve a diverse non-dominated set facilitates the rediscovery of high-throughput layouts under varying interference.

**Figure 9:**
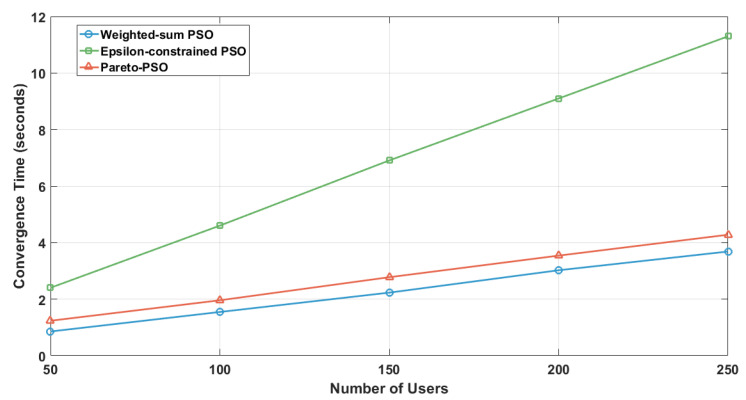
Users number influence on convergence time.

Figure 9 illustrates convergence time as a function of user density for the three optimization strategies. Weighted-sum PSO achieved the fastest convergence, starting at under 1 s with 50 users and rising gradually to about 4 s at 250 users. Pareto-PSO required slightly more time, beginning near 1.2 s and increasing steadily to approximately 4.5 s by 250 users, reflecting the additional computational effort of maintaining and updating a Pareto front. In contrast, Epsilon-constrained PSO showed the slowest performance, with convergence times escalating from around 2.5 s at 50 users to over 11 s at 250 users, indicating that its feasibility checks and repair mechanisms introduce significant overhead as load grows.

Taken together, these results highlight a trade-off between solution quality and computational efficiency. Weighted-sum PSO converges quickly but risks reduced adaptability, Pareto-PSO balances slightly higher computational cost with robust adaptability and superior coverage/throughput performance, and Epsilon-constrained PSO, while theoretically precise in enforcing feasibility, suffers from substantial computational overhead under higher user densities.

**Figure 10:**
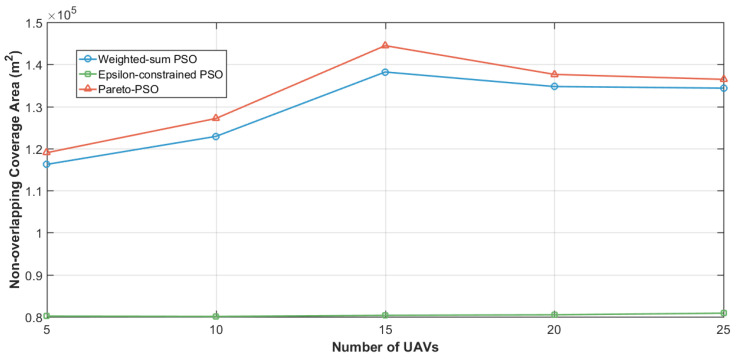
Edge-UAVs number influence on non-overlapping coverage area.

Figure 10 illustrates the scalability of non-overlapping coverage as the swarm size increased from 5 to 25 edge-UAVs. Both Pareto-PSO and Weighted-sum PSO exhibited strong coverage expansion up to around 15 UAVs, achieving peak values of approximately $1.45\;\times \;10^{5}\;m^{2}$ and $1.39\times 10^{5}\;m^{2}$, respectively. Beyond this point, coverage slightly declined due to overlapping service regions and interference effects at higher densities. By contrast, Epsilon-constrained PSO remained nearly flat around $0.8\times 10^{5}\;m^{2}$ across all swarm sizes. Overall, the results confirm that Pareto-PSO consistently discovers the largest non-overlapping coverage envelope.

**Figure 11:**
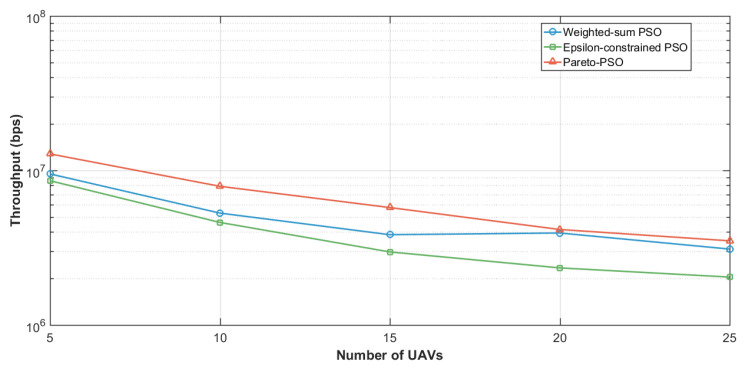
Edge-UAVs number influence on throughput (log Scale Y-Axis).

Figure 11 analyzes the total network throughput as the number of edge-UAVs deployed increased, comparing the performance of Weighted-PSO, Epsilon-constrained PSO, and Pareto-PSO. The results demonstrate that aggregate throughput declines as the number of UAVs increases, reflecting the adverse impact of inter-UAV interference and bandwidth partitioning at higher swarm densities. At five UAVs, all strategies achieved relatively high rates, with Pareto-PSO clearly leading, followed closely by Weighted-sum PSO, while Epsilon-constrained PSO trailed behind. As the swarm size scaled to 10–25 UAVs, the throughput decreased almost monotonically across all approaches, indicating that additional UAVs introduce co-channel contention and resource splitting that reduce the achievable SINR and, consequently, the overall throughput. Despite this decline, Pareto-PSO consistently outperformed the other methods, suggesting that its non-dominated search mechanism reliably identifies deployment geometries and power–altitude trade-offs that mitigate interference effects. In contrast, the Epsilon-constrained PSO remained the lowest performer throughout.

**Figure 12:**
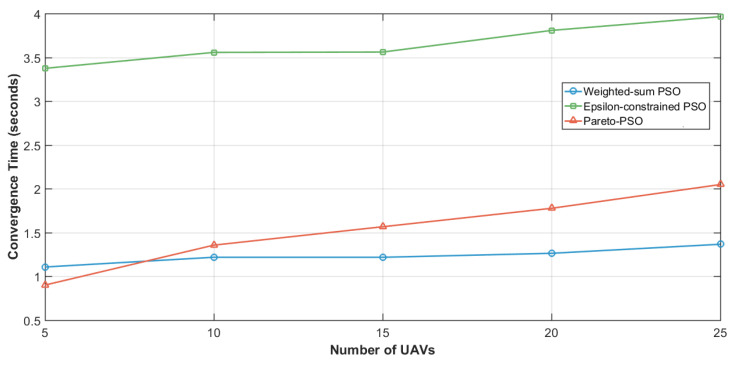
Edge-UAVs number influence on convergence time.

Figure 12 quantifies the scalability of convergence time as the number of UAVs increased from 5 to 25. The Weighted-sum PSO exhibited the lowest sensitivity to swarm size, with its runtime increasing only slightly from about 1.1 s at 5 UAVs to around 1.35 s at 25 UAVs. Pareto-PSO showed a moderate growth trend, rising from roughly 0.9 s at 5 UAVs to just above 2.0 s at 25 UAVs, reflecting the added computational cost of archive maintenance and leader selection as dimensionality expands. By contrast, the Epsilon-constrained PSO consistently reported the highest runtimes, ranging from about 3.35 s at 5 UAVs to nearly 4.0 s at 25 UAVs. Although Pareto-PSO requires slightly higher convergence time compared to Weighted-sum PSO, the difference is not significant, and it still achieves convergence within a very satisfactory time frame. This indicates that Pareto-PSO maintains both robustness and efficiency while providing a richer set of trade-off solutions.

**Figure 13:**
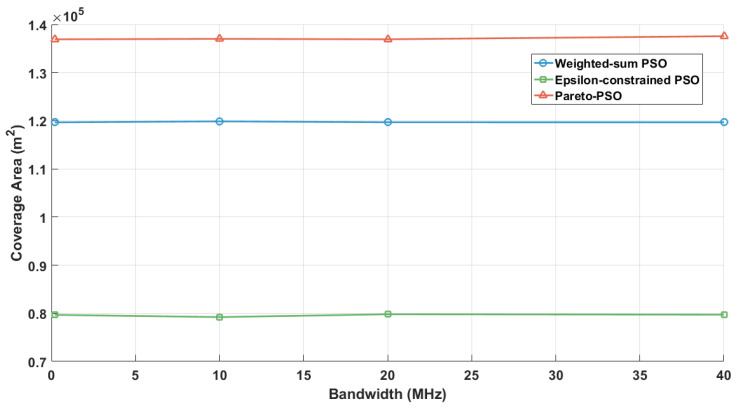
Bandwidth influence on coverage area.

Figure 13 examines the impact of system bandwidth on the achievable non-overlapping coverage for the three optimization strategies. Across the examined range (200 kHz–40 MHz), all curves are nearly flat. This indicates that coverage in the studied system is determined by UAV geometry and line-of-sight conditions rather than spectral width. Among the strategies, Pareto-PSO achieved the highest coverage (≈$1.37\times 10^{5}\;m^{2}$), Weighted-sum PSO performed slightly lower (≈$1.20\times 10^{5}\;m^{2}$), and Epsilon-constrained PSO remained significantly smaller (≈$0.80\times 10^{5}\;m^{2}$). The flat trend across all curves confirms that bandwidth allocation affects throughput but does not expand the physical coverage footprint. Thus, improving coverage should rely on UAV deployment policies such as placement, altitude optimization, and interference management, while bandwidth should be tuned primarily for data rate objectives.

**Figure 14:**
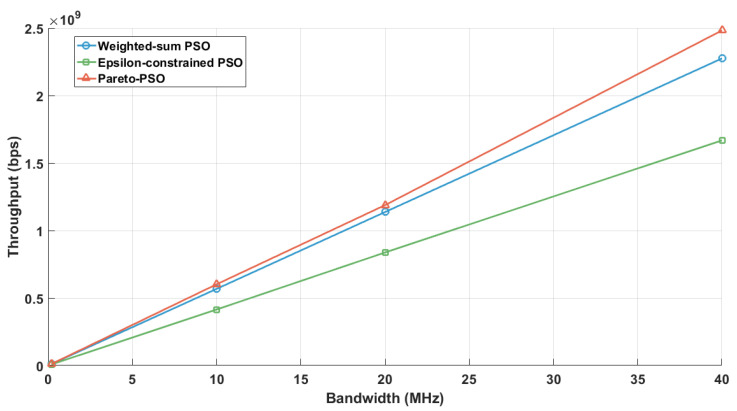
Bandwidth influence on throughput.

Figure 14 depicts a near-linear scaling of the aggregate throughput with bandwidth from 10 to 40 MHz, which is a behavior consistent with a capacity increasing proportionally to the system bandwidth under approximately stable SINR conditions. Pareto-PSO consistently formed the upper envelope, demonstrating superior SINR exploitation and spectrum efficiency. Weighted-sum PSO tracked closely but remained below due to scalarization trade-offs, while Epsilon-constrained PSO lagged, as its feasibility limits restrict aggressive rate maximization. Overall, Pareto-PSO delivered the highest throughput across all bandwidths and achieved steeper scaling, making it the most effective strategy when spectrum expansion is key.

**Figure 15:**
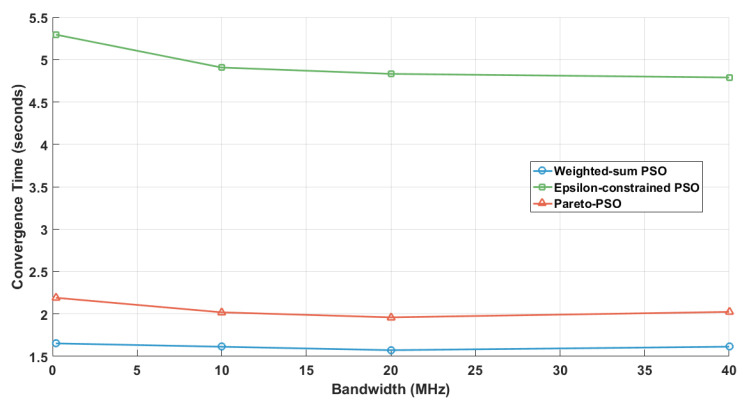
Bandwidth influence on convergence time.

Figure 15 examines solver runtime as a function of bandwidth across 200 kHz–40 MHz, while the number of edge-UAVs was fixed at five, and number of GUs was fixed at 200. Weighted-sum PSO was the fastest and nearly bandwidth-insensitive, fluctuating around 1.6 s, which is consistent with scalar optimization’s minimal overhead. Pareto-PSO showed a shallow U-shape: its runtime decreased from about 2.2 s at 0 MHz to 2.0 s near 20 MHz and then stabilized slightly above 2.0 s, reflecting smoother rate feedback balanced by archive management costs. Epsilon-constrained PSO recorded the highest runtimes but declined from 5.3 s to 4.8 s, indicating that higher bandwidth relaxes feasibility pressure and reduces search effort.

## 6. Conclusions and Future Work

In this paper, we proposed the use of Pareto-based PSO to optimize edge-UAV deployment in the Internet of Flying Things (IoFT). This study demonstrated that the governing factor in edge-UAV deployment performance is the way multiple objectives are mapped into the optimization landscape. By preserving coverage, latency, and energy as a vector of concurrent, non-commensurate objectives, the Pareto-based PSO constructs and maintains a rich frontier that consistently delivers the largest non-overlapping coverage envelope and the highest aggregate throughput across bandwidth levels while sustaining scalable—though moderate—convergence times as swarm size grows. In contrast, fixed scalarization in the Weighted-sum PSO collapses the frontier into a single surrogate objective: this ensures the fastest runtime but systematically tempers extreme gains in coverage and rate whenever the weights diverge from the local frontier geometry. The Epsilon-constrained PSO guarantees explicit feasibility, yet its penalty-driven updates and screening overhead slow convergence and restrict the exploration of aggressively rate-maximizing placements, resulting in weaker throughput. Collectively, these findings confirm that vectorization transforms the optimizer into a frontier builder and selector—retaining decision flexibility for latency-sensitive and energy-aware IoFT deployments—whereas scalarization limits the search to single-point compromises. Consequently, Pareto-PSO emerged as the most effective and reliable strategy for real-time multi-objective edge-UAV deployment, striking a practical balance between throughput leadership, coverage maximization, and acceptable computational cost.

As future work, we aim to compare our proposed Pareto-PSO approach with other multi-objective optimization meta-heuristics, such as genetic algorithms for edge-UAV deployment in IoFT. We will also focus on extending the current decentralized framework into a cooperative distributed model, enabling UAVs to exchange partial solutions and collectively refine global decisions in real time. Future extensions will incorporate resilience tests against UAV loss, link degradation, and environmental perturbations to further validate system reliability. We intend to extend the proposed framework by integrating modern parallel access techniques such as Orthogonal Frequency Division Multiple Access (OFDMA) and other advanced resource allocation mechanisms. These enhancements will allow the model to better capture concurrent data receptions, reduce latency estimates, and improve the framework’s alignment with current UAV communication standards. Incorporating these techniques will also enable a more comprehensive evaluation of network throughput and interference interactions in multi-UAV environments. We will explore the effect of varying weight configurations in the Weighted-sum PSO to evaluate sensitivity and robustness across different priority scenarios, enabling a fairer and more comprehensive comparison of scalarized versus Pareto-based methods. Finally, we will also extend the ϵ-constrained PSO implementation by sweeping multiple ϵ values to generate a richer Pareto front, allowing us to assess the trade-offs between coverage, latency, and energy consumption.

## Figures and Tables

**Table 3:** Simulation parameters.

Parameter	Description	Value
R	Coverage radius	200 m
n0	Noise power spectral density	3.16 $\times \;10^{-14}$ Watts
B	Transmission bandwidth	[2 $\times \;10^{5}$, 10 $\times \;10^{6}$, 20 $\times \;10^{6}$, 40 $\times \;10^{6}$]
S	User data size	randi [2 $\times \;10^{6}$, 10 $\times \;10^{6}$]
numUAVs	Number of edge-UAVs	[5, 10, 15, 20, 25]
numUsers	Number of distributed GUs	[50, 100, 150, 200, 250]
maxIters	Number of iterations	50
numParticles	Number of particles	12
areaSize	Dimension of the simulation area	500 m × 500 m
$\varepsilon_{\mathrm{energy}}$	Energy constraint	4 $\times \;10^{5}$ joule
$\varepsilon_{\mathrm{coverage}}$	Coverage area constraint	4.71 $\times \;10^{4}$ m^2^
$\varepsilon_{\mathrm{latency}}$	Latency constraint	0.8 s
$P_{\mathrm{trans}}$	UAV transmission power	0.5 W
$P_{\mathrm{proc}}$	Edge-UAV processing power	20 W
$P_{\mathrm{travel}}$	Edge-UAV travel power	150 W
$P_{\mathrm{hover}}$	Hovering power	200 W
beta0	SNR constant	1 $\times \;10^{-7}$ W
$W_{\mathrm{coverage}}$	Weight for coverage	0.33
$W_{\mathrm{latency}}$	Weight for latency	0.33
$W_{\mathrm{energy}}$	Weight for energy	0.33
w	Inertia	0.7
c1	Cognition of particle	1.5
c2	Social influence of swarm	1.5

**Table 4:** Performance comparison of Weighted-sum PSO, Epsilon-constraint PSO, and Pareto-based PSO across 10 runs.

Run	W_Cov	Eps_Cov	P_Cov	W_Thr	Eps_Thr	P_Thr	W_Time	Eps_Time	P_Time
1	1.030 $\times \;10^{5}$	8.055 $\times \;10^{4}$	1.322 $\times \;10^{5}$	5.266 $\times \;10^{6}$	4.316 $\times \;10^{6}$	8.144 $\times \;10^{6}$	1.407	3.617	1.913
2	1.357 $\times \;10^{5}$	8.030 $\times \;10^{4}$	1.368 $\times \;10^{5}$	6.944 $\times \;10^{6}$	4.827 $\times \;10^{6}$	6.779 $\times \;10^{6}$	1.164	3.462	1.732
3	1.303 $\times \;10^{5}$	7.840 $\times \;10^{4}$	1.319 $\times \;10^{5}$	6.387 $\times \;10^{6}$	4.695 $\times \;10^{6}$	8.071 $\times \;10^{6}$	1.067	3.256	1.668
4	9.815 $\times \;10^{4}$	8.019 $\times \;10^{4}$	1.223 $\times \;10^{5}$	5.547 $\times \;10^{6}$	4.688 $\times \;10^{6}$	7.066 $\times \;10^{6}$	1.137	3.233	1.615
5	1.233 $\times \;10^{5}$	7.963 $\times \;10^{4}$	1.393 $\times \;10^{5}$	6.638 $\times \;10^{6}$	5.645 $\times \;10^{6}$	9.157 $\times \;10^{6}$	1.103	3.157	1.758
6	1.110 $\times \;10^{5}$	8.078 $\times \;10^{4}$	1.376 $\times \;10^{5}$	7.592 $\times \;10^{6}$	4.849 $\times \;10^{6}$	1.222 $\times \;10^{7}$	1.143	3.351	1.753
7	1.298 $\times \;10^{5}$	8.067 $\times \;10^{4}$	1.367 $\times \;10^{5}$	6.309 $\times \;10^{6}$	5.232 $\times \;10^{6}$	7.426 $\times \;10^{6}$	1.107	3.293	1.725
8	1.265 $\times \;10^{5}$	8.056 $\times \;10^{4}$	1.317 $\times \;10^{5}$	5.474 $\times \;10^{6}$	4.644 $\times \;10^{6}$	6.106 $\times \;10^{6}$	1.075	3.248	1.765
9	1.223 $\times \;10^{5}$	7.876 $\times \;10^{4}$	1.273 $\times \;10^{5}$	6.205 $\times \;10^{6}$	4.272 $\times \;10^{6}$	6.871 $\times \;10^{6}$	1.080	3.340	1.671
10	1.310 $\times \;10^{5}$	7.963 $\times \;10^{4}$	1.362 $\times \;10^{5}$	6.243 $\times \;10^{6}$	4.197 $\times \;10^{6}$	6.800 $\times \;10^{6}$	1.120	3.299	1.551

**Table 5:** Mean and 95% confidence intervals (CIs) for coverage, throughput, and convergence time over 10 runs.

Metric	Weighted-Sum PSO	Epsilon-Constrained PSO	Pareto-PSO
Coverage	$1.220\times 10^{5}\;\left[1.146,1.294\right]\times 10^{5}$	$8.001\times 10^{4}\;\left[7.92,8.08\right]\times 10^{4}$	$1.332\times 10^{5}\;\left[1.288,1.376\right]\times 10^{5}$
Throughput (Mbps)	6.26[5.75,6.76]	4.84[4.60,5.08]	8.26[7.27,9.24]
Convergence Time (s)	1.14[1.09,1.19]	3.30[3.24,3.37]	1.71[1.64,1.78]

**Table 6:** Pareto set of non-dominated solution coverage and latency results at the swarm-level.

Coverage ($m^{2}$)	Latency (ms)	Coverage ($m^{2}$)	Latency (ms)	Coverage ($m^{2}$)	Latency (ms)
4.4271 $\times \;10^{5}$	328.31	4.2965 $\times \;10^{5}$	262.59	4.2701 $\times \;10^{5}$	247.37
4.3204 $\times \;10^{5}$	233.02	4.2688 $\times \;10^{5}$	199.71	4.2884 $\times \;10^{5}$	273.10
4.2743 $\times \;10^{5}$	265.47	4.2713 $\times \;10^{5}$	210.22	4.2819 $\times \;10^{5}$	206.60
4.4416 $\times \;10^{5}$	374.45	3.8750 $\times \;10^{5}$	833.04	4.2951 $\times \;10^{5}$	626.91
4.3913 $\times \;10^{5}$	310.21	3.8638 $\times \;10^{5}$	737.75	3.6980 $\times \;10^{5}$	780.33
4.3865 $\times \;10^{5}$	357.88	4.3531 $\times \;10^{5}$	541.10	3.8593 $\times \;10^{5}$	651.94
3.9121 $\times \;10^{5}$	648.22	3.9205 $\times \;10^{5}$	597.56	4.2750 $\times \;10^{5}$	445.81
3.8159 $\times \;10^{5}$	556.65	3.8213 $\times \;10^{5}$	502.27	4.2480 $\times \;10^{5}$	488.39
3.8952 $\times \;10^{5}$	599.23	4.2477 $\times \;10^{5}$	430.59	4.3513 $\times \;10^{5}$	540.36
3.9969 $\times \;10^{5}$	651.19	3.9595 $\times \;10^{5}$	647.48	3.9447 $\times \;10^{5}$	596.82
4.2589 $\times \;10^{5}$	445.07	3.7466 $\times \;10^{5}$	501.53	4.2892 $\times \;10^{5}$	487.65
4.2420 $\times \;10^{5}$	429.85	4.3951 $\times \;10^{5}$	329.68	4.3886 $\times \;10^{5}$	377.35
4.2357 $\times \;10^{5}$	234.39	4.2849 $\times \;10^{5}$	282.06	4.2853 $\times \;10^{5}$	219.17
4.2714 $\times \;10^{5}$	266.84	4.4169 $\times \;10^{5}$	375.82	4.3359 $\times \;10^{5}$	423.49
4.2499 $\times \;10^{5}$	328.20	3.8577 $\times \;10^{5}$	439.03	3.8712 $\times \;10^{5}$	384.66
4.2635 $\times \;10^{5}$	312.98	4.4094 $\times \;10^{5}$	311.58	4.3846 $\times \;10^{5}$	359.25
4.2619 $\times \;10^{5}$	201.08	4.2585 $\times \;10^{5}$	248.74	3.8075 $\times \;10^{5}$	245.04
4.3852 $\times \;10^{5}$	322.10	4.4063 $\times \;10^{5}$	369.76	4.2691 $\times \;10^{5}$	226.80
4.2470 $\times \;10^{5}$	274.47	4.2853 $\times \;10^{5}$	211.59	4.2579 $\times \;10^{5}$	259.25
3.8430 $\times \;10^{5}$	244.16	3.8464 $\times \;10^{5}$	255.56	4.3584 $\times \;10^{5}$	318.48
4.4024 $\times \;10^{5}$	366.15	4.2666 $\times \;10^{5}$	207.97	4.2380 $\times \;10^{5}$	255.64
4.4083 $\times \;10^{5}$	569.80	3.8697 $\times \;10^{5}$	680.63	3.9225 $\times \;10^{5}$	676.92
4.2247 $\times \;10^{5}$	474.51	3.8038 $\times \;10^{5}$	585.34	3.8406 $\times \;10^{5}$	581.63
3.8625 $\times \;10^{5}$	530.96	4.2561 $\times \;10^{5}$	517.09	3.7709 $\times \;10^{5}$	627.92
3.8332 $\times \;10^{5}$	624.21	3.8561 $\times \;10^{5}$	573.55	4.3788 $\times \;10^{5}$	569.05
3.9320 $\times \;10^{5}$	625.51	4.2861 $\times \;10^{5}$	473.76	3.8884 $\times \;10^{5}$	584.60
4.4369 $\times \;10^{5}$	358.38	4.4092 $\times \;10^{5}$	406.04	4.2712 $\times \;10^{5}$	310.75
4.2713 $\times \;10^{5}$	353.33	4.2901 $\times \;10^{5}$	295.53	4.3663 $\times \;10^{5}$	452.18
4.3112 $\times \;10^{5}$	309.23	4.2822 $\times \;10^{5}$	356.89	3.8569 $\times \;10^{5}$	413.35
4.2383 $\times \;10^{5}$	399.48	4.3982 $\times \;10^{5}$	340.28	4.4582 $\times \;10^{5}$	387.94
4.2965 $\times \;10^{5}$	240.28	4.4315 $\times \;10^{5}$	347.17	4.3051 $\times \;10^{5}$	299.55
4.2436 $\times \;10^{5}$	811.63	4.4006 $\times \;10^{5}$	716.34	4.4441 $\times \;10^{5}$	601.81
4.4050 $\times \;10^{5}$	712.62	4.3441 $\times \;10^{5}$	661.96	4.3504 $\times \;10^{5}$	758.92
4.4100 $\times \;10^{5}$	755.21	4.3196 $\times \;10^{5}$	704.55	4.4885 $\times \;10^{5}$	701.12
4.5265 $\times \;10^{5}$	697.41	4.4280 $\times \;10^{5}$	646.75	4.4250 $\times \;10^{5}$	715.60
4.3961 $\times \;10^{5}$	711.88	4.4563 $\times \;10^{5}$	661.22	4.4399 $\times \;10^{5}$	498.21
4.5086 $\times \;10^{5}$	595.17	4.4870 $\times \;10^{5}$	480.64	4.4441 $\times \;10^{5}$	598.73
4.4069 $\times \;10^{5}$	544.35	4.4984 $\times \;10^{5}$	515.38	4.4190 $\times \;10^{5}$	529.13
4.5660 $\times \;10^{5}$	573.35	4.4914 $\times \;10^{5}$	545.00	4.4967 $\times \;10^{5}$	529.78
4.6377 $\times \;10^{5}$	411.63				

## Data Availability

The dataset can be made available upon request to the authors.

## Abbreviations

The following abbreviations are used in this manuscript:

PSO	Particle Swarm Optimization
UAV	Unmanned Aerial Vehicle
IoFT	Internet of Flying Things
FANET	Flying Ad Hoc Network
IoT	Internet of Things
GU	Ground User
FBS	Flying Base Station
MEC	Mobile Edge Computing
QoS	Quality of Service
IoD	Internet of Drones
GA	Genetic Algorithm
ACO	Ant Colony Optimization
UDA-DD	UAV Deployment Algorithm Degree and Distance
UDA-DS	UAV Deployment Algorithm Degree and Signal-to-Interference-plus-Noise Ratio
DVFMC	Distributed Virtual Force Motion Control
ELAPSE	aErial Local Positioning System for Emergency
GPS	Global Positioning System
MILP	Mixed Integer Linear Programming
TTT	Total Travel Time
GAGLPP	Ground–Air-Controlled Global and Local Path Planning
LOS	Line-Of-Sight
NLOS	Non-Line-Of-Sight
QoC	Quality of Coverage
DRL	Deep Reinforcement Learning
SINR	Signal to Interference plus Noise Ratio
mmWave	millimeter-Wave
IAB	Integrated Access and Backhaul
DCEE	Dual Control for Exploitation and Exploration
RRT	Rapidly exploring Random Tree
UL-NOMA	UAV-enabled upLink Non-Orthogonal Multiple Access
PSO-VRFFA	Particle Swarm Optimization–Virtual Repulsive Force Firefly Algorithm
PSO-GA-G	Particle Swarm Optimization-Genetic Algorithm-Greedy
MOP	Multi-objective Optimization Problem
DSLPSO	Double Self-Limiting Particle Swarm Optimization
SLRMSA	Self-Limiting Radius and Multiple Simulated Annealing
PF	Pareto Front
MOPSO	Multi-Objective Particle Swarm Optimization
